# Proton transfer reactions: From photochemistry to biochemistry and bioenergetics

**DOI:** 10.1016/j.bbadva.2023.100085

**Published:** 2023-03-09

**Authors:** Alexander P. Demchenko

**Affiliations:** a Yuriy Fedkovych National University, Kotsubynskoho 2, Chernivtsi, 58012, Ukraine; b Palladin Institute of Biochemistry, Leontovicha 9, Kyiv, 01030, Ukraine

**Keywords:** Proton transfer dynamics, Biochemical reactions of proton transfer, Proton transfer photochemistry, Light-activated reactions, Proton channels, Photosynthesis and cellular respiration

## Abstract

The present Review is an attempt by projecting the basic knowledge on photochemical proton transfer to achieve consistent understanding of proton motions in biocatalysis, photobiocatalysis, operation of selective proton channels and systems of photosynthesis and cellular respiration. The basic mechanisms of proton transfer are in active research in the electronic excited states of organic molecules. This allows observing the reactions directly in real time, providing their dynamic and thermodynamic description and coupling with structural and energetic variables. These achievements lay the background for understanding the proton transfers in biochemical reactions, where such ultrafast events are not only ‘optically silent’ but are hidden under much slower rate-limiting steps, such as protein conformational changes, substrate binding and product release. The mechanistic description of biocatalytic and transmembrane proton transport is shown as a multi-step proton migration that is available for modeling in photochemical reactions. For explaining the formation of transmembrane proton gradients, a simple ‘proton lift’ concept is presented that may be the basis of further research and analysis.

## Highlights

• The mechanisms of proton transfer established in photochemical reactions in organic dyes are discussed in order to achieve deeper understanding of functioning of biochemical systems.
• From this focus, the proton motions in biocatalysis, photobiocatalysis, operation of selective proton channels and systems of photosynthesis and cellular respiration are analyzed.
• For explaining the formation of transmembrane proton gradients, a simple ‘proton lift’ concept is presented that may be the basis of further research and analysis.

## Graphical abstract

**Figure:**
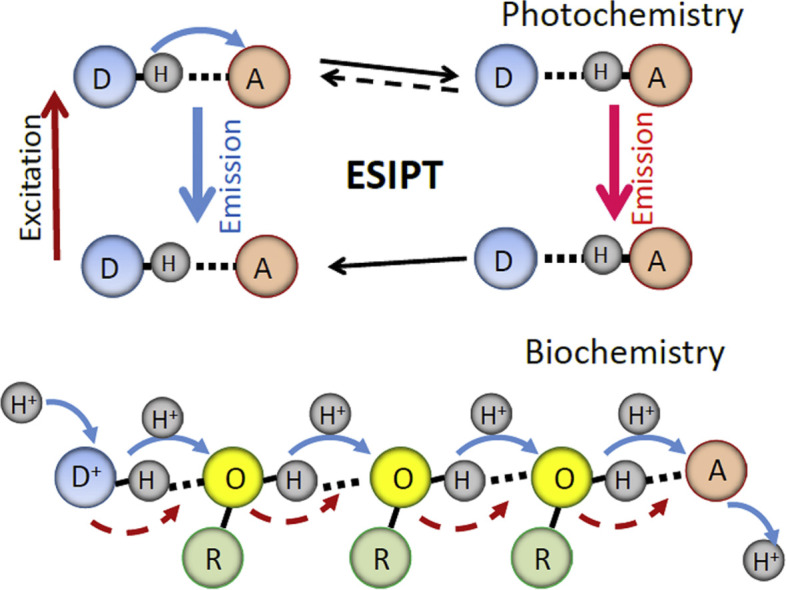

## 1 Introduction

Proton transfer (PT) [1], [2] is one of the basic reactions that are of ultimate importance for different branches of chemistry [3], [4], [5], [6] and biology [7], [8]. Its fundamental role [7], [9] in biochemistry is demonstrated in almost all chemical reactions catalyzed by enzymes [10], [11], operation of proton channels [12] and generating proton gradients in the energy transduction machinery [13]. The mechanisms of these reactions in living systems are difficult to harness experimentally due to intrinsic complexity of the considered systems and to their coupling with other reaction steps proceeding slower and being commonly the rate-limiting, such as substrate binding and product release that may be coupled with protein conformational changes, their associations, binding inhibitors, etc. [14]. Known among the fastest reactions in chemistry, the PT elementary steps are commonly by orders of magnitude faster than the overall rates observed in kinetic experiments that record consumption of reactants or accumulation of reaction products. It would be ideal to study the PT steps on their own time and energy scales, but such possibility exists only for a very limited number of reactions that can be activated by light quanta and allow collecting information in the form of light emission. The very important experimental tools available to biochemists are still rather modest and commonly are presented by analysis of structural data with atomic-scale resolution, directed mutations, pH titrations, observations of isotope effects and the studies of steady-state and transient reaction kinetics that do not allow accessing the ultrafast elementary reaction steps. It is essential that in proteins the overall conformation may provide substantial constrains on interactions between atoms and their groups that are critically important for PT reactivity; they may lead to dramatic energetic effects being indistinguishable in X-ray crystallographic structures [15].

The lack of direct general experimental accessibility to observation of basic PT steps in biocatalysis and proton transport suggests looking at simplified systems that could allow deriving their inherent mechanisms together with estimation of their kinetic and thermodynamic variables and the factors that influence them. Such possibilities exist. They are offered by organic dyes performing the excited-state intramolecular proton transfer (ESIPT) reactions [16], [17] that possess the ability to light-up the “dark” events by generating the informative fluorescence emission.

**Fig. 1:**
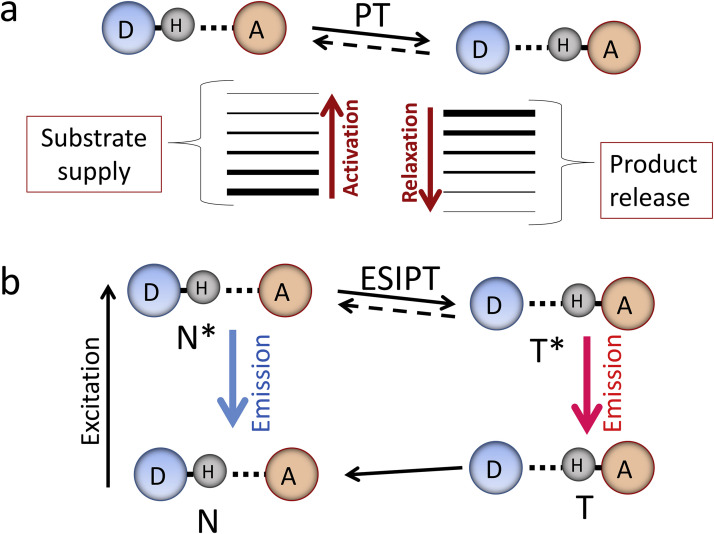
Comparison of the ground-state chemical or biochemical PT reaction and the excited-state intramolecular proton transfer (ESIPT). (a) The PT step in the ground-state transformation. Reaction starts after the donor-acceptor coupling with the H-bond by thermal activation of vibrational modes. The product is released or it participates in coupled reactions. The release of product is an indicator of the reaction rate. (b) The ESIPT reaction. The intramolecular H-bond exists but the thermal energy is insufficient for overcoming the reaction barrier. Here both reactant and reaction product can be highly fluorescent, so that the temporal depopulation of reactant and population of the product can be observed directly. The reaction starts from the supply of energy of electronic excitation to normal N state, and the emissions from initial N* and product T* excited states allow observing its occurrence, rate and reversibility in real time. The reaction product after decay to the ground T state is transformed to initial N state in a ground-state back reaction.

The similarity and differences between the ground-state PT reaction and ESIPT are illustrated in Fig. 1. In both cases, the reaction can occur only on the condition of existence of H-bond coupling the proton donor and acceptor, and its basic mechanisms are the proton tunneling or activated over-barrier proton motion [18]. The involvement of vibrational modes associated with H-bonds driving the reaction to activated state and the polarization of solvent (protein) environment that responds to the motion of charges may be needed in both cases [19], [20].

Essential differences are in the following. In the ground-state, the formation of reactive state is critical and it is often achieved by diffusing and proper binding of reactants. This may be the slowest step limiting the overall reaction rate and hiding the PT step. Reaction kinetics is commonly followed in experiment by the release of reaction product or by its involvement in a coupled process that can be also the rate limiting. The closed catalytic cycles can be constructed in the ground states but they will contain slow steps and, in most cases, the informative response should be observed as the slow consumption of substrate or release of reaction product. Therefore, in the studies of these reactions the scientist has to be satisfied by static information or by the kinetics determined by the slowest step.

In contrast, in ESIPT the electronic excitation starting the reaction is the fastest step. The light quanta power the ultra-fast thermodynamically uphill processes without any additional input of other forms of energy. The short light pulses start the reaction in ensemble of reactive species in synchronized manner and allow following the development of reaction in time for this synchronized ensemble. The informative response can be obtained in the form of fluorescence emission on the real (often, ultrafast) time scale from both reactant and reaction product. Therefore, the ESIPT reaction allows observing the PT process in real time being not obscured by any side effects but with the broad possibilities of imposing and analysis of these effects. This allows observing the reaction in different regimes with the determination of all kinetic and, in some cases, thermodynamic parameters [21]. Possessing these tools, one can obtain information of fundamental value that should be common for photochemical and biochemical systems.

Many ties unite photochemistry and biochemistry. This connection is seen in the basic mechanisms of photosynthesis [22] and photoreception [23]. Photoenzymes use the light energy for biochemical transformations [24], whereas the light generated in biochemical events [25] serves for communication between different living organisms. The author's prediction on possibility of simulation of biochemical events with photochemical reactions [26] have found many ways for realization. Current literature contains a number of attempts to model the specific biological events with organic dyes exhibiting ESIPT reactions. By exploiting their excited-state properties and their dynamics, the abilities are found to impose the light-controlled electronic, energetic and structural requirements for selective and efficient chemistry of such biocatalyst models. This new strategy already achieved some success in creating small functional molecular enzyme-like systems and resulted in artificial photoenzymes operating in the absence of specific protein environment [27]. Inspired by the knowledge of performance of biological systems, the new electrocatalysts have been developed [28].

The other trend, which is the simulation of photophysical processes occurring in biological systems with simplified models, is also in active development. The examples that can be noted here are the studies of double PT in dimers mimicking this reaction in complementary DNA bases [29] and the PT reaction in green fluorescent protein (GFP) chromophore [30]. However, even with the knowledge of individual protein structure and function and with the ability to identify its reactive site, such simulations look naïve if they are not based on some general principles that could be in the basis of biological proton transfer. I believe that such principles can be derived from the results of ESIPT studies on organic dyes. In this direction, some important aspects of biology-related PT reactions will become clearer, such as the reaction driving force and the activation barrier, the role of strength and geometry of H-bond connecting the donor and acceptor groups, and also the involvement of pre-organization and dynamics of protein structure surrounding the reaction sites and proton-conducting networks.

The recent advances in understanding the ESIPT reactions in organic dyes and of the mechanisms of action of different intrinsic and extrinsic factors allowed formulating a system of empirical rules [17], [31] that, I believe, are basic for all proton transfer reactions including that occurring in biological systems. The aim of the present Review is to translate the regularities obtained in experiments on organic dyes to the world of biochemistry and to show their importance as the models for obtaining new knowledge that allow comprehending basic PT events in biochemistry and bioenergetics.

## 2 Proton in chemical and biochemical transformations

The role of proton in chemical and biochemical reactions is great and specific. Proton is by ∼1836 times heavier than the electron and in different reactions it behaves both as classical atom and as a quantum-mechanical object [32]. Because of its much heavier mass, its wavefunction falls off with distance much steeper than the electronic wavefunction, by ≈ 40 times. This means that in any chemical, photochemical or biochemical system, a one-step PT is possible only in the case of very close location of donor and acceptor, in their direct contact and commonly on their coupling with hydrogen bonds (H-bonds) [2]. Meantime, different bioenergetic, biocatalytic, and membrane transport reactions require the transport of protons for much larger distances. As we will see below, this is realized only by coupling of multiple elementary steps along the pre-organized pathways.

### 2.1 Proton transfer – electron transfer coupling

Because of high reactivity and ability to bond formation, proton in any condensed system does not exist alone but is always a part of molecular structure. Moreover, when it is bound to electronegative atom, such as oxygen or nitrogen, it can form an H-bond with another atom possessing a lone pair of electrons. It is known that the H-bond energy and its length on sub-Ångstrom scale [33] should influence critically the PT reactions. However, when these reactions proceed as intermolecular events, such as in protic, hydrogen-bonded liquids, the donor-acceptor distances are large. In water, the O-H...O distances are typically around 2.85 Å. From an energetic perspective, the closer distances seldom occur in solutions, because the van der Waals repulsion exceeds electrostatic attraction, and the intermolecular mobility creates the distribution of distances and orientations of the partners. In proteins (as well as in model ESIPT performing dyes) the situation is different. Their reactive geometries are more or less fixed and the configuration of H-bonds can be constrained [34], which allows realizing O-H...O distances on a much shorter lengths scale (∼2.4–2.7 Å) [35]. The theory predicts that even so short equilibrium positions of H-bonded atoms are not optimal for PT, they should be closer [36], which suggests that the reactive PT events can be possibly achieved by coupling with vibrations [20].

In contrast, the wavefunction of electron is broad, propagating at significant distances, up to several nanometers. Moreover, the ET proceeding as an activated process or by tunneling demonstrates its rate decreasing exponentially with the distance, and this decrease does not depend strongly on surrounding structures [37]. Despite these differences, being of opposite charges, proton and electron participate in different reactions in a coupled way. The coupling of ET and PT allows reducing the reaction free energy cost by excluding the motion of charged species. This allow realizing the broad range of ET-PT reactions depending on the strength of this coupling and its spatial range.

**Fig. 2:**
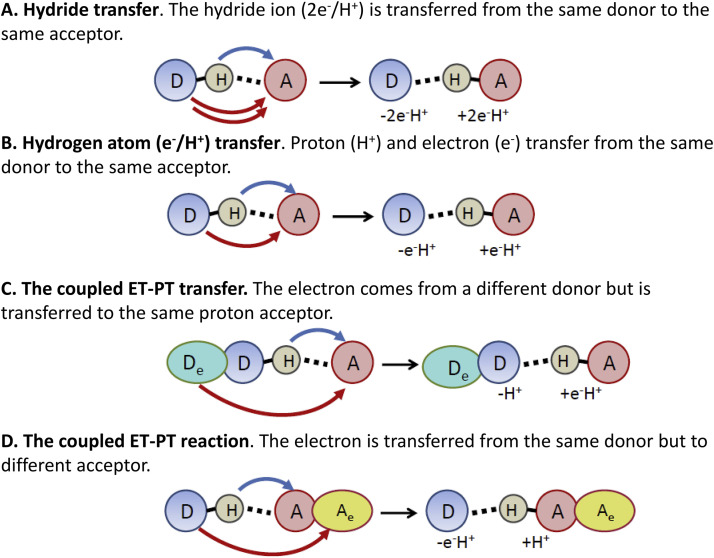
The basic reactions involving the coupled transfer of proton and electron. In hydride transfer reaction, the hydride ion (2e^−^/H^+^) is transferred from the same donor to the same acceptor and is typical for redox enzymes (Case A). Hydrogen atom (*e*^−^/H^+^) transfer occurs when the proton (H^+^) and electron (*e*^−^) transfer occurs from the same donor to the same acceptor (Case B). The coupled ET-PT transfer in which the electron comes from a different donor but is transferred to the same proton acceptor in such a way that transfer of electron occurs to compensate the charge separation that appears in the reactant state (Case C). The coupled ET-PT reaction, in which the electron is transferred from the same donor but to different acceptor, generating the charge-separated state (Case D). Blue arrows show the motions of protons and red arrows of electrons. The donors are D for protons and D_e_ for electrons. A and A_e_ denote the correspondent acceptors.

Since proton transfer and electron transfer are different by the nature, rate, distance, the involved masses, etc., they exhibit very different properties. Meantime, their coupling of diverse types can be observed in nature (Fig. 2).

In hydride transfer reactions (such as in NAD(P)H oxidoreductases, [38], [39]), an excess electronic charge is transferred together with proton. When the proton and the electron are transferred simultaneously from the same donor to the same acceptor with no charge effects, these reactions are commonly called the hydrogen atom transfers [40]. In the other extreme case of such coupled reactions, an electron can move a long distance in one direction while the proton moves only at a very short distance in the same or orthogonal way. Such motions of electrons can create the charge transfer (CT) states, up to full charge separation.

The ET-PT coupling can be of variable nature and strength. There is a continuous discussion whether in the excited-state reactions the two ET and PT transfers proceed concertedly or they follow a sequential stepwise pathway [41], [42], [43]. In the first case, the active motion of proton and electron follows along collective reaction coordinate for PT and ET. In contrast, in the latter case the first reaction precedes and promotes the second one, so that the two events are coupled although temporally separated. Meantime, even in photochemical systems the rigorous, well-defined characterization of these processes as sequential or concerted was not achieved [16]. The diversity of opinions about that is because these reactions are extremely fast and they do not allow catching and characterizing the intermediates [11], [44]. Here we note that ET-PT coupled reaction can be concerted and of zero activation energy, even if it proceeds from the same donors to different acceptors [45], the case in Fig. 2,d.

Sequential ET-PT reactions are not favorable because of formation of the reaction intermediates that, being the high-energy charge-separated states, should result in strongly reduced rates. Therefore, many researchers consider the concerted mechanism that utilizes the overall free energy change in a single reaction step. However, the analysis of such reactions is a great challenge for the theory. If both reactions follow the Marcus-type non-adiabatic (weak electronic coupling) description [46], the probability of the coupled reaction is very low because of necessity to tunnel simultaneously both an electron and proton in a vibration-coupled manner. Therefore the descriptions are developed to consider one of these reactions to be non-adiabatic, and the other − adiabatic (strongly electronically coupled) [11]. This allowed consistent treatment of elementary steps of chemical and biological ET-PT reactions [47].

As the smallest ESIPT performing systems, organic dyes are attractive for focused theoretical studies [11], [36]. Mutual enrichment of theory and experiment has led to important advancement in understanding the coupling of electron and proton movement. Such coupling of proton transfer (PT) and electron transfer (ET) denoted as ET-PT is often called the proton-coupled electron transfer (PCET) [40], [47].

### 2.2 Proton transfer observed in steady-state and time-resolved domains

Chemical and biochemical reactivity is conventionally described in broad terms of kinetic versus thermodynamic control [21], [48]. Classically, the lowest activation barrier among the various reaction paths determines the kinetic control, whereas the relative free energies of the reactant and the final products determine the thermodynamic control. This principle can be applied to complex multi-step reactions for determining their overall yields and rates. The excited-state PT reactions monitored by fluorescence techniques provide the only presently foreseen experimental way to observe the broad range of elementary PT events directly, with ultra-high resolution in time and energy and with the possibility of analysis of their energetics and kinetics in terms of reaction mechanisms [21].

**Fig. 3:**
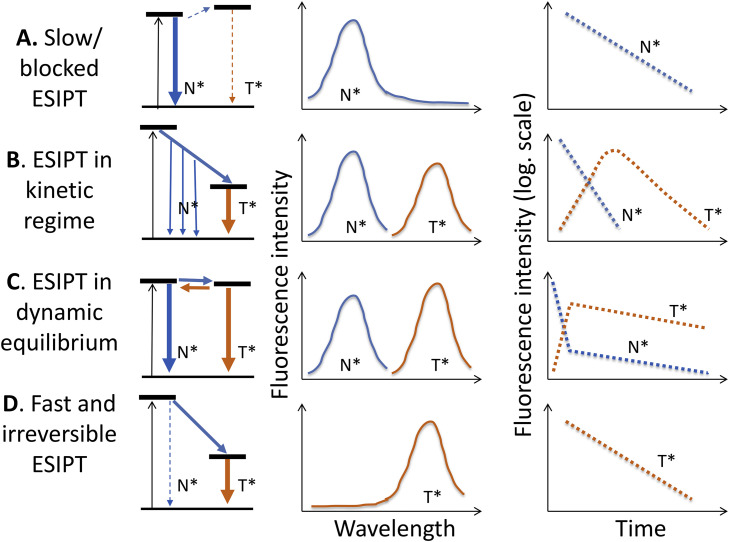
Illustration of different kinetic regimes in ESIPT reactions. Simplified energy diagrams, steady-state spectra and time-resolved decays are presented. (A). The reaction does not proceed or is very slow compared to the fluorescence lifetime. Emission from the N* band in steady-state spectral (left) and time-resolved (right) formats is observed only. (B). The reaction proceeds in kinetic regime. Both N* and T* bands are observed in steady-state fluorescence spectra. The time-resolved decays demonstrate the fast N* component and simultaneous growth of the T* component that subsequently decays with its characteristic lifetime. (C). The reaction proceeding in thermodynamic regime. Both N* and T* species are present in fluorescence spectra. The equilibrium is established rapidly with the decay of N* emission and rise of T* emission, and then, at longer times, both emissions decay with the same rate. (D). The reaction proceeding in kinetic regime is fast and irreversible. Only the T* emission is observed in recorded spectra and emission decays. The time window that allows observing fluorescence kinetics is determined by the excited-state lifetime, which for different ESIPT fluorophores occupy picosecond (10^−12^ – 10^−10^) and early nanosecond (10^−9^ – 10^−8^) time range.

The fluorescence bands of ESIPT dyes are commonly located in the visible range of spectrum and are well resolvable. The reactant is the initially excited normal (N*) form demonstrating fluorescence band at higher energies (shorter wavelengths) and the product tautomer (T*) form with the band at lower energies (longer wavelengths). These reactions are reversible in a sense that passing through the whole cycle involving the excitation to N*, the following ESIPT reaction to T*, the T* emission and the ground-state back transformation to N state, the system appears again in the basic ground state (Fig. 1b). Furthermore, in excitation-PT-emission-back transfer cycle the accumulation of reaction product does not occur, and the reaction can be performed over and over again with the same species. Numerous experiments have demonstrated that by variation of molecular structures of ESIPT dyes and of their environments, different situations depending on the interplay of reaction kinetics and fluorescence decay kinetics can be realized (Fig. 3):

Let us consider these cases in more detail analyzing the energy diagrams, steady-state spectra and emission decays presented in Fig. 3.

(A) The excited-state PT reaction does not occur or occurs very slow on the time scale of the excited state lifetime (commonly, nanoseconds). There may be several reasons for that, such as perturbation by external H-bonding, stabilization of polar N* state in polar environment or slow conformational change in flexible dye that puts together the ESIPT partners. In this case, the steady-state and time-resolved emission from the N* state is observed only.
(B) The PT reaction is moderately slow and proceeds within the time range of fluorescence emission. This is the case of ESIPT reaction with an energy barrier. In this case, both N* and T* bands are simultaneously present in the steady-state emission spectrum. In the time-resolved decays one may observe the decrease of intensity of the N* band and its increase for the T* band with the same rate. The decay that follows proceeds with different, usually slower, rate.
(C) The PT reaction is ultra-fast but reversible. Then the equilibrium between reactant (N*) and product (T*) forms is established on a time scale much faster than the emission. Both N* and T* bands are observed in the steady-state emission spectra and the ratio of their intensities characterizes the dynamic equilibrium between these forms. However, the time-resolved kinetics is different. Establishment of N*↔ T*equilibrium can be seen as the fastest components of emission. It is negative (decaying) for N* form and positive (appearing) for the T* form. Then the fluorescence from N* and T* forms starts to decay with the same longer rates.
(D) The PT reaction is ultra-fast and kinetically irreversible. In this case, the N* state is depopulated so rapidly that the appreciable amount of emission from this state cannot be detected in the steady-state spectrum that contains only the T* band. Only in the time-resolved spectra with ultra-high (fs) resolution, the depopulation of the N* band can be observed.

The presently available time-resolved fluorescence techniques allow approaching very broad time windows covering the femtosecond, fs (10^−15^–10^−12^ s), picosecond, ps (10^−12^–10^−9^ s) and nanosecond, ns (10^−9^–10^−7^ s) ranges. The ultra-short laser pulses can synchronize the reaction by establishing time zero and then following its development in time. This allows studying on the scale of reaction kinetics the effects of different factors, from molecular vibrations (in fs range) to dielectric relaxations in highly viscous media (ns range) [16]. Also, since the reactions proceed in the excited state, additional possibilities can be realized, such as manipulation with the input energies that allows the excitation to high-energy electronic states or high vibrational states [49] and variation of input power (the density of excitation quanta) resulting in highly populated reactant states and non-linear effects [50].

### 2.3 Basic regularities in excited-state proton transfer reactions

The majority of biochemical PT reactions involve strong H-bonds formed by O–H type proton donors. These reactions, though they are the most popular in photochemical ESIPT studies, are also the most difficult to research in kinetic regime. Typically they are nearly barrierless and highly exergonic (releasing the energy) giving solely a proton-transfer tautomer emission with sub-picosecond rise time (Fig. 3,d), and only by providing the charge-transfer substitutions the reaction is switched to thermodynamic regime (Fig. 3,c). In contrast, the systems with N–H proton donor groups are rare, and an interest to them in biology is related to tautomerization of DNA bases in double-helical structures [51], [52], [53]. However, due to much weaker H-bond strength and the possibility of variation the proton donor acidity by applying electron donor and acceptor substitutions at the same proton donor site, they allow the broad-scale modulation of the ESIPT energetics and rate. This can be done by replacing in proton-donor nitrogen an H atom that does not participate in ESIPT for a variable substituent –R to form the N(R)-H donor groups [54]. The inclusion of the electron-withdrawing substituents increases the acidity of the amine, as well as the polarization of the N–H bond and the H-bond donor strength. In this way, an impressive result was obtained for 2-(2′-aminophenyl)benzothiazole dye. By small chemical substitutions, the ESIPT reaction was transformed from ultrafast to slow and ultraslow regime.

**Fig. 4:**
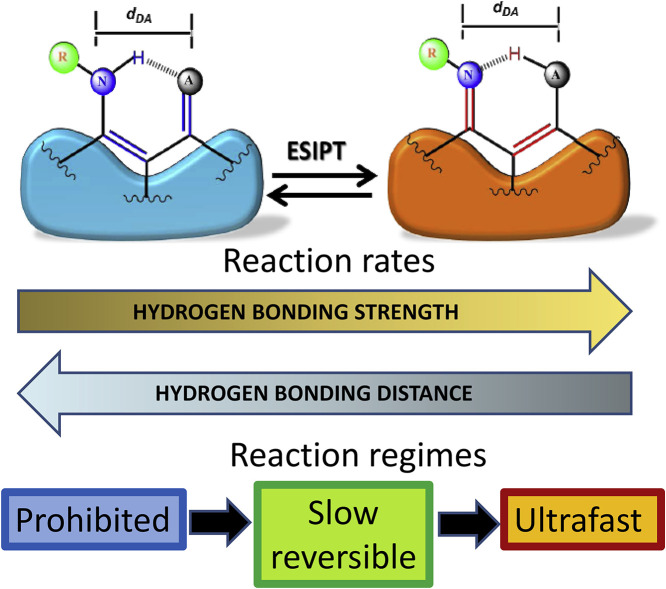
Illustration of dramatic variation of the reaction rates of ESIPT reaction as a function of H-bond distance and strength. The results obtained in the series of PT dyes possessing the N(R)–H type donors demonstrate that with the variation of H-bond strength the reaction shifts from being prohibited to slow reversible and then to ultrafast [17], [54] demonstrating sequentially the spectroscopic and time-resolved features depicted in Fig. 3.

Since the systems with N(R)-H donors demonstrate much slower ESIPT reaction rates, this allows their focused studies with common time-resolved techniques. Therefore, one can systematically fine-tune the dynamics of N(R)–H ESIPT systems as a function of H-bond strength by varying the electronic properties of the –R substituent. The ESIPT rates became easily measurable as well as their response to different applied factors, from dye structures to solvent and temperature. As a result, the switching between different thermodynamic and kinetic regimes was clearly observed, and the correlations among the donor-acceptor distances, proton acidity, H-bond strength, and the ESIPT kinetics and thermodynamics were established [17]. They are visualized in Fig. 4 and summarized as empirical rules below.

(1) **The excited-state acidity of the proton donor** produces the strongest effect on the occurrence and rate of ESIPT reaction. By its variation in molecular design, the switching of the reaction thermodynamics from endergonic to exergonic (producing the reaction driving force) can be generated [54]. This allows achieving three reaction regimes, i.e., prohibited, thermodynamic (slow reversible) and ultrafast, without or with minimal perturbation from the environment.
(2) **The higher H-bond strength** accelerates the ESIPT reaction. This fact can be explained by the H-bond induced stronger π-delocalization between proton donor and acceptor, leading to decrease of the energy of reaction product T* state relative to the N* state energy, i.e. increasing the ESIPT exergonicity. In this respect, the ultrafast reactions observed with O–H donors [16] may be considered as the limiting cases, in which the factor of high H-bond strength is the strongest.
(3) **The activation barrier** ΔE_a_ decreases with closer H-bond distance. Accordingly, it decreases linearly with a decrease in the T* state energy, making the reaction more exergonic. Thus, in analogy to Marcus electron-transfer theory [46], the relationship in ESIPT between reaction dynamics and thermodynamics becomes evident. This result requires closer look at an interplay between the activated proton transfer and its tunneling. In this respect, it is known that the activated processes commonly proceed adiabatically, and in this case their probability can be much higher than that of proton tunneling [55]. Therefore, the existing concepts of ESIPT mechanism as the reaction proceeding without a barrier [56] are not always correct, and the intrinsic barriers retarding the reaction may exist.
(4) **The conformation and its rigidity** of the H-bonding partners play a crucial role in establishing and modulation of ESIPT reaction regime. Optimizing these factors by selecting the rigid six-membered configuration [57] over the flexible seven-membered ring system [58] accelerates ESIPT. In contrast, in systems requiring structural planarization (e.g., involving strictly localized zwitterion-like character), the ESIPT reaction must be retarded [59]. An additional factor that can be involved here is the decrease of activation energy due to the reduced excited-state structural rearrangement resulting from stronger hydrogen bonding.

The rules formulated above are valid both in solutions and in solid state [17]. They got confirmation in the studies of systems with O-H proton donors [60]. It can be stated that the PT studies in the electronic excited states capture the elementary nature of proton transfer that is of general value for chemistry and biochemistry.

### 2.4 Examples of realization of intramolecular, complexation-assisted and solvent-assisted proton transfer

Several selected examples presented below demonstrate the diversity of small-molecular systems for closer modeling the proton transfer in biology.

**Fig. 5:**
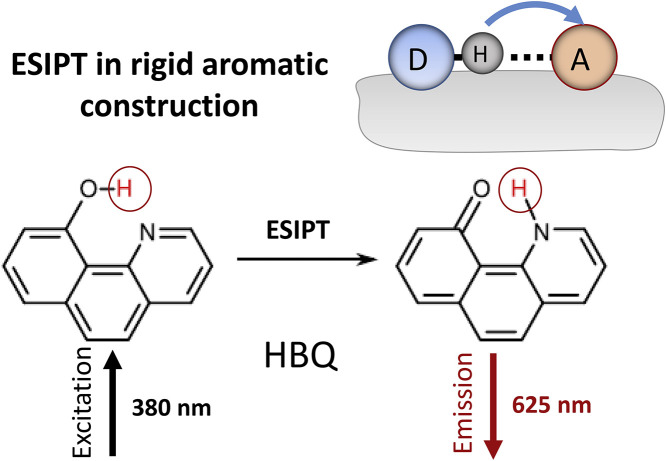
The ultrafast ESIPT reaction without barrier and solvent perturbations. 10-Hydroxybenzo[*h*]quinoline (HBQ) as an example.

The ability to demonstrate the ultra-fast ESIPT reaction kinetics with the observation of only the emission from T* state (case D in Fig. 3) can be realized in different dyes. Optimal conditions for that are rigid skeleton of molecule with optimal geometry and high strength of H-bond that is the ESIPT pathway. There are many examples of this behavior that can be considered classical [4]. One is 10-hydroxybenzo [h] quinolone [61], see Fig. 5. It displays unidirectional ultra-fast (10^−14^–10^−12^ s) ESIPT kinetics driven by increased acidity of the donor and basicity of the acceptor, so that a single-exponential emission decay kinetics and single spectrum of T* form are observed.

It was concluded [61], [62]) that ESIPT reaction in HBQ is essentially barrierless and proceeds through a single-well mechanism. Its rate may be coupled with the period of low-frequency large-amplitude vibrations incorporating the motion of atoms associated with the hydrogen bond. This dye possesses perhaps the strongest hydrogen bond (estimated to be ∼10 kcal/mol) as the result of geometry restriction, which forces the optimization of hydrogen-bonding distance and orientation [60]. Due to strong intramolecular hydrogen bonding, this emission is free from external H-bonding perturbation, even in water.

**Fig. 6:**
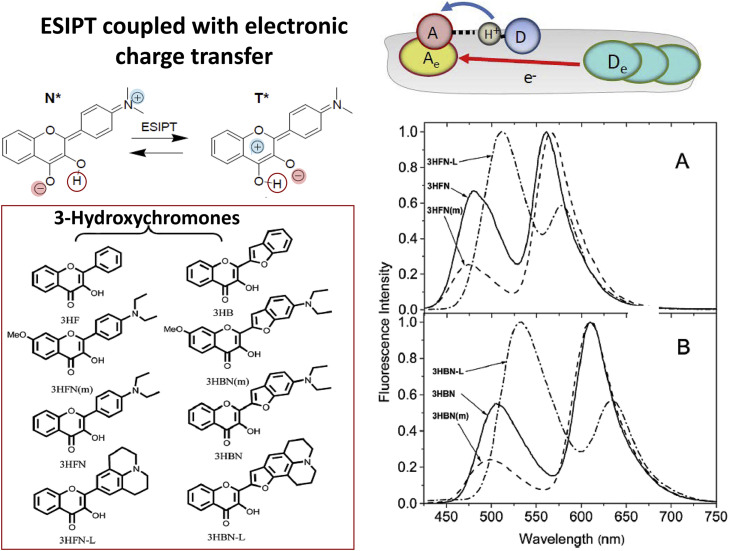
ESIPT in the case of variable to strong charge-transfer character of the reactant excited state. Typically, reverse reaction repopulating the reactant state is productive, leading to excited-state equilibrium. Two series of 3-hydroxychromone dyes based on dialkylamino-substituted phenyl or benzofuranyl at position 2, both exhibiting dramatic variation of λ-ratiometric response to polarity of molecular environment [63], [64] are presented. (A) The two-band fluorescence spectra of flavones 3HFN, 3HFN-L and 3HFN(m) in chlorophorm and (B) benzofurylchromones 3HBN, 3HBN-L and 3HBN(m) in toluene. Excitation at 420 nm. The variation of substitutions change the strength of electron donors, which modulates the ESIPT reaction.

The extent of coupling between proton transfer and the transfer of electronic charge can provide dramatic variation in behavior of ESIPT performing dyes [16]. Just by providing chemical substitutions with electron donor or electron acceptor groups, one can change between kinetic and thermodynamic regimes (cases B and C in Fig. 3), induce or suppress sensitivity of ESIPT to various external factors, such as local polarity, external H-bonding or electric fields [21]. Modulating substituents in parent ESIPT dye by attachment of electron-donating or electron-withdrawing groups change drastically the influence of electronic polarization on this reaction and on its spectroscopic behavior. Such generation and modulation of ET-CT coupling in ESIPT reactive dyes allows observing the effects of appearance or disappearance of strong electric dipole. Its interaction with the environment (surrounding groups of atoms) induces their molecular motions (relaxations). The studies of time-resolved and steady-state spectra provide unique possibility for determining molecular interactions and their dynamics, characterizing the local polarity and local electric fields [16]. Those are the aromatic molecules based on 3-hydroxychromone core [65], [66], [67] (Fig. 6).

Different biochemical PT reactions involve several elementary steps. As the models, in the focus of researchers can be different organic molecules that possess two electronically coupled ESIPT sites. Two or more ESIPT reactive sites can be realized within the same organic heterocyclic molecule with the possibility of studying the coupling of their reactivities. Direct interaction between protons in such systems is not possible a priori, and the possibility of coupling may be realized by conjugation between electronic sub-systems responding to each proton-transfer step. Different extent of π-electronic conjugation can be realized in a single molecule modulating the proton transfer. Such systems were designed and investigated in detail by both steady-state and time-resolved techniques [68], [69], [70]. In their studies, both concerted [71] and sequential [72] double transfers were demonstrated.

**Fig. 7:**
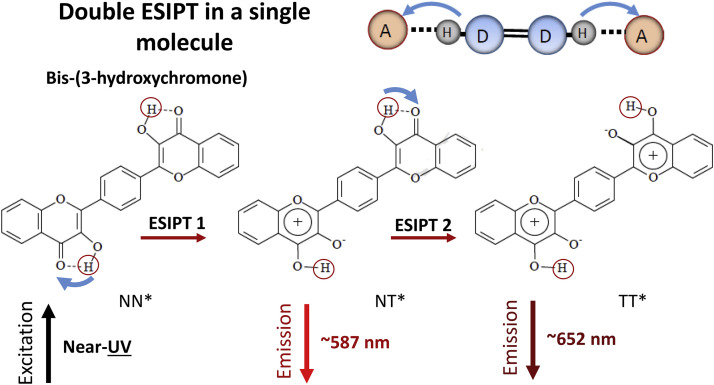
Bisfavonols as the models of two electronically coupled ESIPT reactions. These reactions can occur both simultaneously and sequentially. An example is the 3-hydroxy-2-[4-(3-*hydroxy*-4-*oxo*-4*H*-2-*chromenyl*)*phenyl*]−4*H*-4-chromenone molecule, in which the chromone moieties are conjugated directly via the lateral aromatic rings [70].

One of such systems contains two mutually symmetric and identical intramolecular hydrogen-bonded 3-hydroxyflavone fragments [70]. They are bound with two methyl spacers to *para*-positions of the same benzene ring (Fig. 7). Interacting on photoexcitation, they undergo either single or double proton transfer that are distinguished by the wavelength of their emission.

**Fig. 8:**
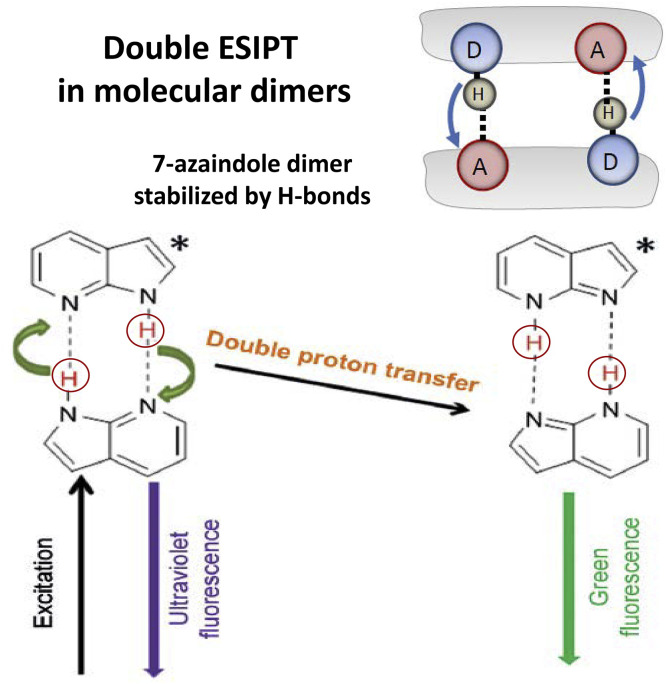
Double ESIPT in molecular dimers. Each monomer has the PT donor and acceptor group, so that two H-bonds support the dimer structure and are the ESIPT pathways.

Of special interest as the models of biochemical PT reactions are the systems, in which these reactions proceed in molecular associates with close approach of donor and acceptor. Strong acceptors (e.g. amines) can form the H-bonded complexes with the dyes in solutions [73]. One of special cases that got popularity in photophysical studies are the coupled H-bonded dimers, so that the donor of one molecule is the acceptor for the other (Fig. 8). Their most studied example is the 7-azaindole H-bonded dimer [74], [75] that undergoes a switch of two symmetric protons in the excited state, denoted as excited-state double proton transfer (ESDPT). Such systems were suggested as the models of dual proton transfer between complementary bases in double-stranded DNA that protects it from photodamage induced by UV radiation [29].

The idea of coupling between the excited-state proton transfer units assembled within a single organic dye molecule is interesting in view of a prospect for developing the “proton wires” modeling the operation of biomembrane proton channels [76]. A step to its realization could be a molecule with two or more electronically coupled proton-transfer units, in which structural and energetic asymmetry could make this transfer directed. The issue of concerted (one-step) and/or sequential (two-step) pathways can be resolved in these systems.

**Fig. 9:**
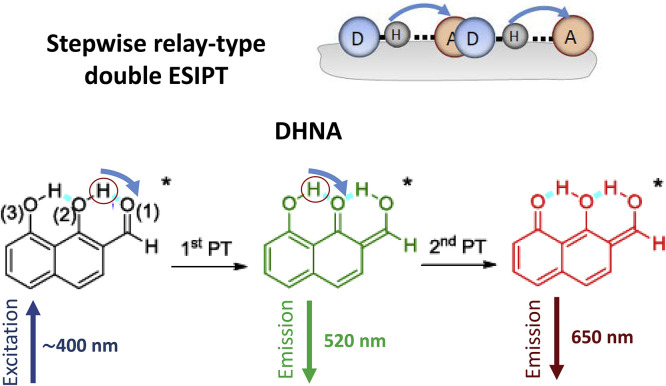
Stepwise directed double proton transfer in the excited state of 1,8-dihydroxy-2-naphthaldehyde (DHNA) possessing double intramolecular hydrogen bonds [72].

Possessing double intramolecular hydrogen bonds, 1,8-dihydroxy-2-naphthaldehyde (**DHNA**, Fig. 9), and its derivatives represent the case of stepwise, relay-type intramolecular double-proton transfer in the excited state [72]. Upon excitation, two large Stokes shifted emission bands with maxima at 520 and 650 nm are resolved when studied both in cyclohexane and in single crystal. They were attributed to the tautomer emission resulting from the first and second proton-transfer products. The first proton transfer is ultrafast, whereas the second reaction in sequence is reversible and proceeds on picosecond time scale.

When the potential proton transfer donor and acceptor within the same molecule do not contact directly, there is a possibility to connect them by “proton wires” formed of bridging molecules with the H-bonded linking. Water [77], [78], organic acids [79] and alcohols [80], [81] that possess the ability of a single oxygen site to accept and donate protons may act as the proton wires [9].

The characteristic examples of multiple molecular wires connecting donor and acceptor sites in organic dyes are the 7-hydroxyquinoline (7HQ) derivatives that in the presence of a H-bond bridging diol in a polar aprotic medium form a reactive cyclic H-bonded 7HQ–(diol)_2_ complex [81], [82]. It was shown that in bulk water two mechanisms of proton transfer could be realized. One is by diffusion of hydrated proton and the other, more efficient, by proton wire [78]. The rate-determining step in bulk water or alcohols is the slow solvent reorganization to form the cyclic H-bonded complexes. In nonpolar aprotic solvent, these complexes formed by added bridging molecules are more stable and provide more efficient PT [83].

**Fig. 10:**
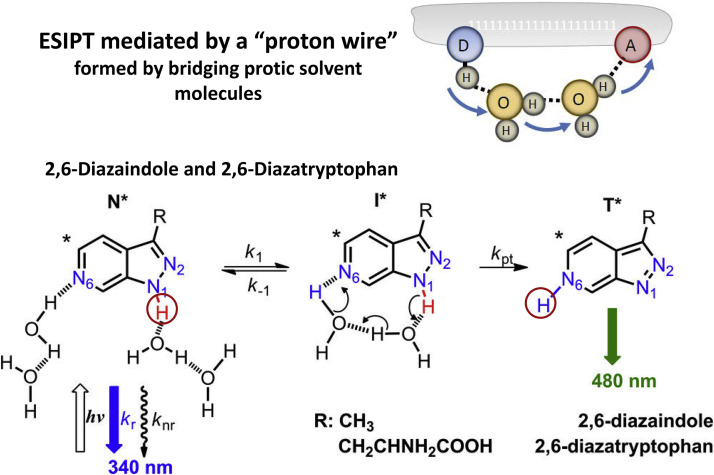
The proton wires connecting ESIPT donor and acceptor sites that are too far apart to donate/accept a proton directly. In 2,6-diazaindole and 2,6-diaza-Trp, the ESIPT reaction is mediated by dynamic formation of proton wires by two bridging water molecules [84].

The ESIPT bridged with a single water molecule was demonstrated for synthetic Trp-analogs, 2,7-diazaindole and 2,7-diaza-Trp [80]. Whereas their normal emission is observed in the near-UV range, a new fluorescence band appears in the visible range due to such bridging. When the donor-acceptor distance is larger, such as in 2,6-diazaindole and 2,6-diaza-Trp, the bridge can be formed by the arrangement in chain of two water molecules [84] (Fig. 10). Interestingly, the cell synthetic machinery is able to recognize these molecules as natural Trp-molecules and to insert them into desired protein sites. Combination of fluorescence responses of inserted 2,7-diaza-Trp-and 2,6-diaza-Trp-residues was suggested for studying the water-associated conformational mobility in the active sites in proteins [85].

The possibilities for efficient modeling of multi-step proton transfers through H-bonded chains (proton wires) are of special interest, since these reactions are realized in green fluorescent protein and in different enzymes and ion channels. Presented above examples demonstrate that designing of more advanced models that impose free energy gradients in asymmetric PT wires with possibilities of studying different energetic and conformational variables is a reality.

Thus, intramolecular, intermolecular and solvent-bridged excited-state PT reactions allow broad range of possibilities for modeling the chemical and biochemical PT events. The systems can be found for mechanistic studies of different intramolecular, dimeric and solvent-assisted excited-state reactions. Their driving force comes from the input of energy on electronic excitation. The excitation triggers the change in the distribution of π-electron density of the molecules that results in CT-PT coupling generating an increase in acidity of proton donors and/or in basicity of proton acceptors. Such arrangement (or rearrangement) of electronic sub-system may not be necessarily intramolecular but extended to larger distances. The PT reaction site can be assembled from different, e.g. enzyme-substrate, components connected by H-bond. This bond should be relatively strong and attain the configuration favorable for the reaction.

### 2.5 Understanding the effects of ESIPT reaction medium

The proton transfer reactions in chemistry and biochemistry are exquisitely sensitive to a number of factors, including pH, electrostatics, proper active-site geometry, and chemical design [11], which provides different possibilities for their control and regulation. The most sensitive to these factors is the electronic sub-system that determines the energetic and kinetic variables for proton transfer reactions. In photochemistry, these processes are studied by recording the dynamic Stokes shift, i.e., the frequency down shift of the fluorescence spectrum following optical excitation. In these studies one must consider the extended system that involves the surrounding molecules (or their groups of atoms) that respond to the change in electric field produced by electron and proton motion.

**Fig. 11:**
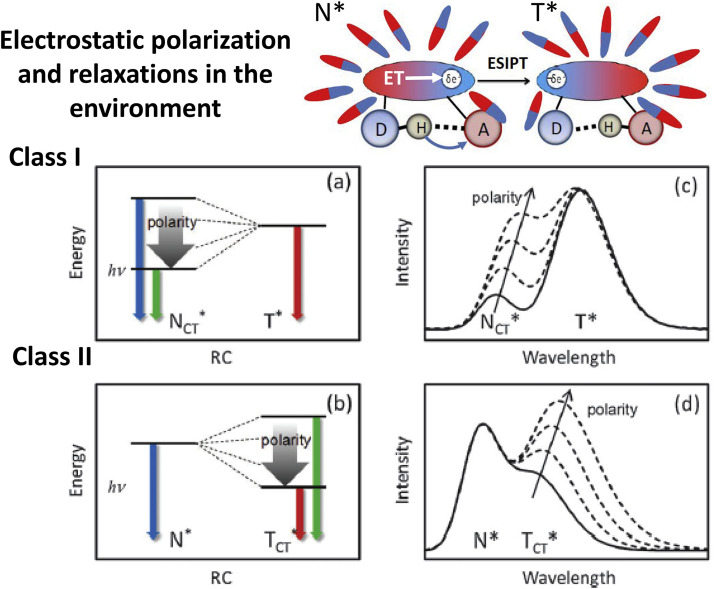
Two limiting cases of coupled excited state CT–PT reactions [16]. (a) CT first, producing the charge transfer N_CT_* state with variable energy depending on interaction with dielectric environment. In T* state the dipole moment disappears and its energy is solvent-independent. (b) PT first, generating the charge transfer in the product T_CT_* state. Its energy becomes variable and dependent on intermolecular interactions/relaxations. The corresponding steady-state spectra illustrating the CT/PT dynamics are presented as (c) and (d) respectively. Dashed lines in (c) and (d) denote the expected spectral change subject to solvent polarity change and molecular relaxations and the arrows indicate the direction of these changes.

This response generates an appreciable solvent-induced barrier and thus transforming ultrafast ESIPT to proceed on a much slower time scale. The surrounding responds to the motion of charges by electronic polarization and relocation of surrounding dipoles and charges [86] and, rarely, by more extended conformational changes. In organized protein environment, these relaxations are restricted and may be extended to nanosecond and longer times [26]. Fluorescence is the popular method of studying these dynamic processes occurring in the direction of attaining the dynamic equilibrium, the relaxations. The most significant effects on spectra, especially in polar media, are produced by relocation and reorientation of dipoles surrounding the ET-PT performing dye, as shown in Fig. 11. This process depends strongly on the dye properties, particularly on the way of coupling between electron and proton motions. It was suggested [16] to categorize the ET-PT systems into two classes:

**Class I** molecules, in which the proton acceptor and electron acceptor happen to be in the same group, but the electron donor and proton donor sites are different.
**Class II** molecules, in which the proton donor and electron donor are indistinguishable, but the proton acceptor and electron acceptor sites are different.

In both cases, the significant solvation perturbation effect on ESIPT dynamics is observed, but the difference is in formation of CT states, if it occurs before or after ESIPT. In former case, the large dipole moment appears in reactant N* state resulting in strong solvent-dependent wavelength shifts of correspondent fluorescent band. Synthetic means allow by simple chemical substitutions to achieve modulation [64], [87], [88] or even reversal [89] of the dye excited-state dipole moment with respect to PT coordinate. Such strong reorganization of the electronic sub-system results in transformation of emission from ultrafast (Fig. 3,d) to the reversible thermodynamic (Fig. 3,c) regime [21]. Modulated by the solvent generating reaction barriers, the forward and reverse reaction rates can be shifted to much longer times, to tens of picoseconds [90]. The reaction is very sensitive to external perturbations demonstrating an interplay of intensity between N* and T* emission bands as a function of such external factors as solvent polarity [16], [91] or local electric fields [92]. Characteristic representatives of Class I dyes are 3-hydroxychromones with their various electron-donor substitutions (see Fig. 6).

The Class II dyes possess quite different properties. The ESIPT reaction can proceed only in kinetic regime. The primary electronic excitation does not generate strong dipoles and, therefore, fluorescence from N* band is solvent-independent. Dipole moments appear as a result of distribution of electronic charge in the course of ESIPT reaction. Therefore, kinetics of dielectric relaxations is different. The position of T* band becomes dependent on solvent polarity (Fig. 11).

**Fig. 12:**
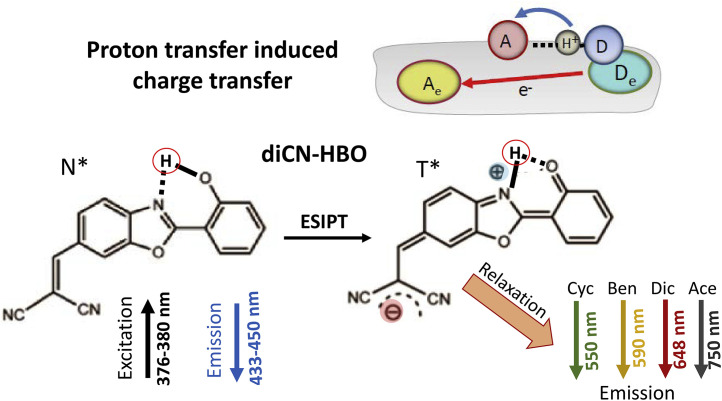
The photochemistry of benzazole dye diCN—HBO that demonstrates coupling of ESIPT with an electron transfer to remote acceptor [93]. In N* state the electronic charge separation is insignificant, and fluorescence spectra are solvent-independent. ESIPT reaction brings substantial charge separation, so that in the T* state the dye becomes the strong dipole interacting with solvent molecules. As a result, the emission from T* state strongly shifts to longer wavelengths as a function of solvent polarity. The fluorescence band maxima are indicated for cyclohexane (Cyc), benzene (Ben), dichloromethane (Dic) and acetonitrile (Ace).

The benzazole dye diCN—HBO is an example of Class II dyes (Fig. 12). It undergoes ESPT, concomitantly accompanied with the charge transfer process, such that the ESPT reaction dynamics are directly coupled with solvent polarization effects [93]. The long-range solvent polarization interactions result in a solvent-induced barrier that retards the overall proton transfer reaction rate. The solvent-dependent fluorescence spectra extend to extremely broad range, from green to near-infrared.

Thus, we possess organic dyes that were strategically synthesized as the tools for deeper understanding the basic PT mechanisms and for providing versatile studies in selected environments. The present experience with them includes probing the PT in different media, from gas phase [94], liquid crystals [95] and supercritical fluids [96] to solids [62], [97], [98], nanoscale aggregates [99] and cryogenic matrices [100]. Efficient are the studies of proteins with dye attachment by site-specific binding [101] or covalent labeling [102]. Popular are the studies of cell membranes [103] and their lipid models [104]. Moreover, ESIPT dyes can serve as artificial DNA base analogs that provide information on their site-specific interactions [105]. These reaction environments can modulate the directionality and rate of ESIPT reactions in broad ranges.

## 3 General features of proton transfer in biochemical reactions

There is a major longstanding interest in elucidating the fundamental mechanisms in the background of the enormous catalytic proficiencies of enzymes, of the efficiencies and selectivities of ionic channels, and of the pathways for biological transformation and utilization of energy. Proton transfer plays a key role in these reactions.

### 3.1 Proton-transfer abilities of protein reactive sites

The basic mechanisms of proton transfer reactions described above for typical ESIPT dye molecules should be valid for the reactions involving PT steps in biocatalysis and transport. In most of the studied cases this reaction is represented as a coupled ET-PT [106], which is similar to that observed in well-investigated organic molecular models [16]. However, the structural realization of these mechanisms is different [107]. Several aminoacid residues, particularly Tyr, His, Trp, and Cys, are employed as ET-PT carriers and mediators together with the often present substrates, ligands and cofactors incorporated into the protein structures [44].

Here Tyr (pK_a_ = 10.1) and Cys (pK_a_ = 8.2) that readily dissociate protons are the key players. At physiological pH values, the Tyr-oxidation is associated with deprotonation of the phenolic oxygen, giving rise to ET-PT reaction. The protein structures with the redox-active Tyr-typically include associated His-base and, for Cys-oxidation, an Asp-carboxylate base [14], [106]. Water bridges are also found [108] and the same role as in photochemical PT reactions (see Section 2.4) was attributed to them. Covalently coupled or strongly H-bonded constituents of these reactions allow construction of different molecular models.

The Tyr-residues function in different proteins as the intermediates that are transiently oxidized and reduced during the long-distance electron transfer [44], [109], [110]. When the proton is transferred to a His-residue in hydrogen-bonding contact with the participation of electron accepting function, the neutral tyrosyl radical can be formed avoiding the appearance of a high-energy acidic phenolic form. Such tyrosine-based ET-PT reactions are found in photosystem II (see Section 7.2), which carries out the light-induced oxidation of water [111] and in ribonucleotide reductase, which reduces ribonucleotides to form deoxynucleotides (see Section 4.4). It was shown that in compounds modeling the TyrOH—His-pair the pendant bases or solvent molecules act as proton acceptors [112].

**Fig. 13:**
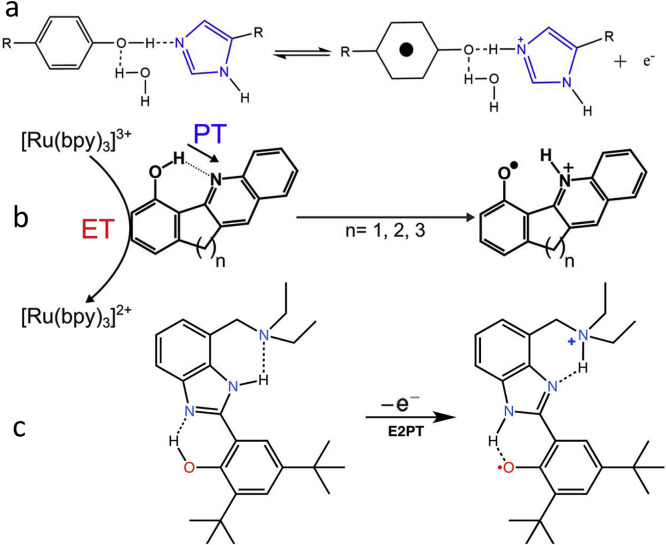
The modeling of ET-PT reaction in the tyrosine-histidine pair that is basic for Photosystem II function. (a) The reaction between phenol and hydrogen-bonded imidazole derivative, in which oxidation of phenol is accompanied with the protonation of an imidazole group [109]. (b) The model that allows estimating the H-bonding length between the phenol oxygen (proton donor) and quinoline nitrogen (proton acceptor) by variation of covalent bridge via 5, 6, or 7 membered carbocycle [118]. (c) A conjugate of substituted derivatives of benzimidazole and phenol with a secondary proton acceptor (tertiary amine) that is hydrogen bonded to the distal NH of the benzimidazole. Upon electrochemical oxidation of the phenol, the system undergoes two concerted proton transfer reactions, resulting in a phenoxyl radical and an ammonium ion [119].

Fig. 13 illustrates the proton coupled electron transfer (ET-PT) reactions between the H-bonded phenol and imidazole derivatives as the analogs of Tyr-and His, in which the oxidation of the former is accompanied with the protonation of the latter. Also schematically presented are other simplified models that, among many others, can be useful for obtaining detailed information about the fundamental properties that govern these reactions and characterizing them as concerted one-electron two-proton transfers (see also [113], [114], [115], [116]). In a recent study [117] this pair was covalently attached to a porphyrin sensitizer. Using the laser flash photolysis it was found that in the presence of an external electron acceptor the water molecules were needed to gate the movement of both the phenol and imidazole protons along an isoenergetic pathway.

**Fig. 14:**
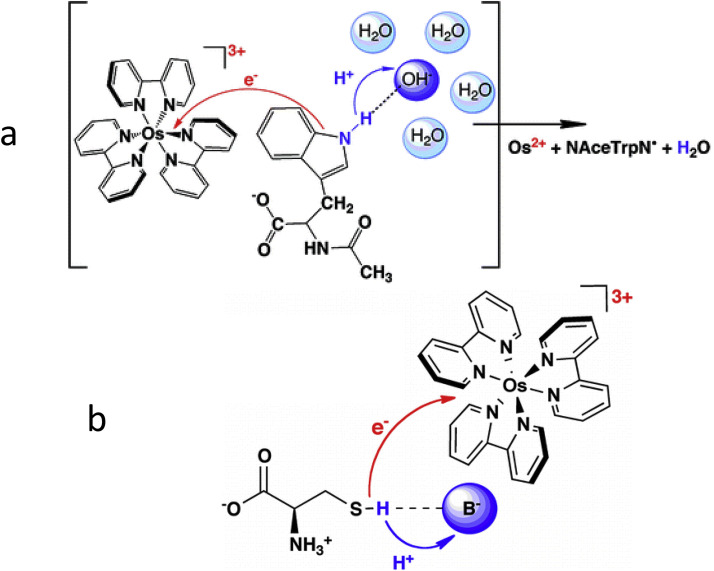
Oxidation of amino acids in a coupled ET-PT reaction. (a) Tryptophan [120]. (b) Cysteine [123].

The biologically important oxidation of tryptophan is the ET-PT reaction with proton transfer to water [120]. Being a secondary amine (pK_a_ ≈ 16–17), tryptophan, in contrast to tyrosine, can donate proton only to a very strong acceptor, such as in azurin [121]. However, the transfer of electron to such strong acceptor may decrease dramatically its pK_a_ value [120]. This case is presented for the coordinated osmium compound, see Fig. 14,a. In this reaction, the OH^–^ group in water but no other common bases can serve as proton acceptors. Based on results with the application of oxidants generated by laser flashes in aqueous solutions, it was stated that the proton transfer to water is a general feature of amino acid oxidation in ET-PT reactions that can be assisted by proper electron acceptor [122].

Cysteine with p*K*_a_ = 8.2 is the most acidic of the three common redox active amino acids. The stop-flow and electrochemical measurements were consistent to demonstrate its ET-PT reaction in concerted manner [123] as shown in Fig. 14,b. Oxidation is rate-limited by initial 1e^–^ electron transfer to M(bpy)_3_^3+^, with multiple added proton acceptor bases.

**Fig. 15:**
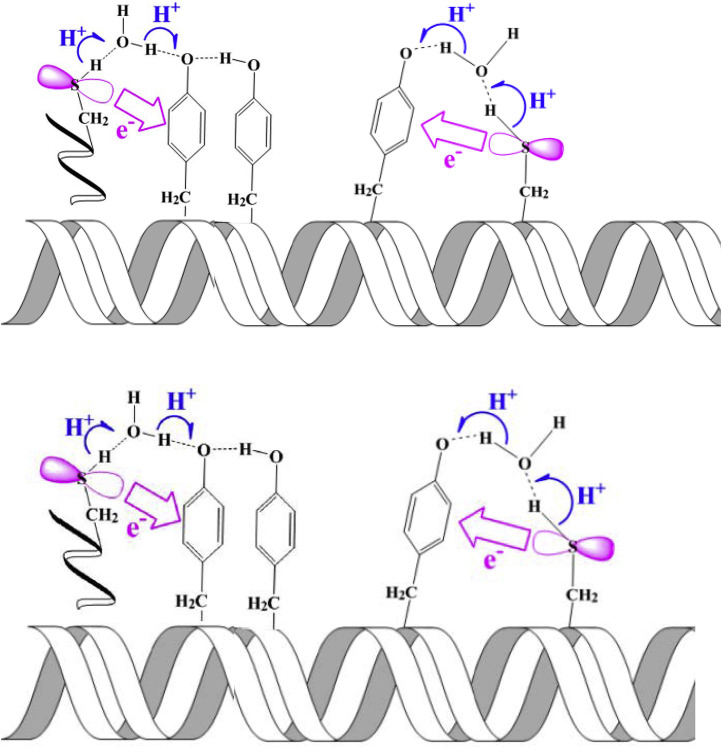
The mechanisms for the proton/electron transfer reactions from Cys-to Tyr• in proteins suggested on the basis of DFT and QM/MM calculations [108]. The double ET-PT can be realized in the cases when a water molecule inserts between Cys-and Tyr• side chains.

Direct PT-ET reaction between Cys-residue and tyrosinyl radical (Tyr•) is an important step for many enzyme-catalyzed processes (e.g. class I ribonucleotide reductase). The *ab initio* calculations on simplified models and quantum mechanical/molecular mechanical (QM/MM) calculations on real protein environment reveal that direct electron transfer between them is difficult to occur, but an inserted water molecule can greatly promote the proton/electron transfer by a double-proton-coupled electron transfer mechanism [108]. The inserted H_2_O molecule links the side chains of Tyr• and Cys-via two H-bonds and acts as the proton relay. It also enhances the electronic overlap between the lone-pair orbital of sulfur atom and the π-orbital of phenol moiety and thus functions as the electron transfer pathway (Fig. 15).

**Fig. 16:**
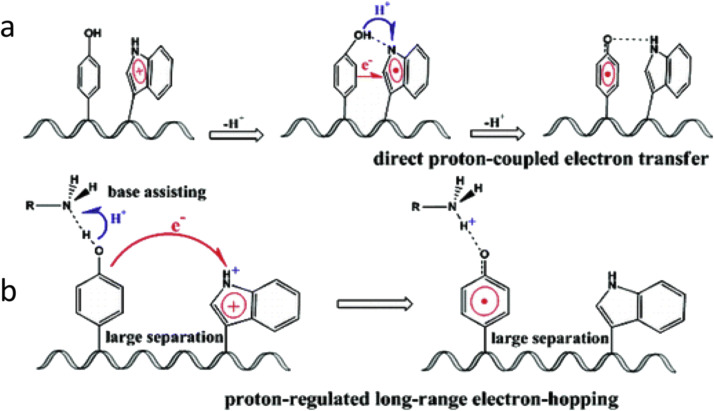
Suggested mechanisms of ET-PT from Tyr-to Trp-radical cation in a series of synthetic peptides [124]. (a) Direct transfer involving Tyr-and Trp-only. (b) Long-distance hopping ET coupled with PT to a closely located hydrogen bonded base.

The ET-PT reactions between Trp-and Tyr-residues play an essential role in the long-range electron transfer involved in signaling and enzyme function. Examples of this type of reactions are found in ribonucleotide reductase [7], see Fig. 20. These reactions have been studied using DFT calculations and *ab initio* molecular dynamics simulations [124]. The authors considered several cooperative transfer mechanisms from a Tyr-to Trp-radical (or cation) without or with the assistance of a base and suggested that different reaction regimes can be realized in proteins. They range from direct proton-coupled π-electron π-channel/σ-channel transfers to proton-coupled long-range electron hopping, depending on the distance in protein structure (Fig. 16).

The His-residues being frequently protonated in proteins at physiological pH play a special role as proton donors in different biological PT reactions [125], [126]. Their imidazole group is analogous to a carboxyl containing two atoms (N_d_ and N_1_) that can be protonated. However, because of the absence of intramolecular H-bond, proton cannot hop between these two sites without assistance of coupling groups, e.g. by forming the H-bond wires. The combination of His-and acidic residues is a motive, which is found in cytochrome c oxidase (see Section 7.3) and in bacterial photosynthetic reaction centers. It is able to capture protons from the aqueous phase for providing them to reaction sites of proton-transfer proteins and proton channels [127].

The understanding of such PT and coupled ET-PT reactions in water is even more challenging, since water can act as a proton acceptor or donor. Moreover, pK_a_ of an amino acid inside a protein can be substantially different from that in free solution, depending on the polarity and local electric fields in their environment [128]. The protein conformational transition may change this condition dramatically. This appears to be an important factor modulating PT in catalytic mechanisms of carbonic anhydrase II [129], the voltage-gated proton channels [130] and other systems.

Proton acquisition and proton release are separable events for an acid or base, and the switching between them modulated by local electrostatics can be achieved by rotation of a single amino acid side group. Such switch is described for the carboxyl group at the entrance to the D channel for proton transfer of cytochrome c oxidase [131]. This group accepts a proton from solvent water, then rotates donating proton to the first water in the channel (see Section 7.3). Such carboxyl rotation may be common for modulating the proton transfer pathways in proteins.

In active research are the oxidative-reductive properties of model systems composed of small molecular compounds of biological interest, such as NAD(P)H, flavines, metal-porphyrin complexes and their analogs and also of antioxidants such as ascorbate, tocopherol derivatives and of transition metal ions. The results of their studies are well described in literature [132] and are not discussed here.

### 3.2 The hydrogen bonded proton transfer relays

In different biological PT reactions, proton travels a relatively long distance through the membrane or to/from a reaction site [11]. Meantime, both the theory and experiment show that there is no way for proton to move in one step at a distance longer than the H-bond length [97]. The only possibility for realizing a long-distance proton transfer is to provide it in multiple steps along the formed H-bonding network. Therefore, the proton channels are so constructed that each group on a pathway can accept a proton from a donor and release it to an acceptor located within the hydrogen bonding distance [133], [134]. The donor-acceptor intermediates can consist of aminoacid side groups with possible involvement of water molecules.

Water has unique property to be the smallest donor and acceptor of proton [135], [136]. It can form hydrated proton structures in solutions [137], and its ability to provide bridges between PT donor and acceptor groups located distantly in organic dyes was discussed in Section 2.4. It can pass through the protein channels [22] and be the integral components of protein structures [138]. Moreover, a cooperativity in strengthening the H-bonded water network was found, so that adding a new molecule to the chain provides local strengthening of the whole chain [139]. In this research, electrochemical method was applied to study the compounds, in which the covalently coupled OH group is inserted between potential proton donor and acceptor, and forming the donor-acceptor network allowed the PT reaction to proceed [140]. These experimental observations add weight to our understanding of possible operation of H‐bonded chains in proteins that are able to transfer protons.

The direct measurements of PT rates offered by time-resolved spectroscopy (see Fig. 3) become valuable for providing information on the reaction mechanism in terms of energetic and kinetic variables. They show that the proton migration over substantial distance is fast, much faster than the diffusion of water molecules themselves [141], which can be seen in the results of femtosecond time-resolved studies [78]. However, the coherent proton tunneling may not operate on moving along the chain coupled by H-bonds due to very small probability of quantum mechanical coupling between all the constituting elements. Moreover, if the proton moves as a positive charge, it should polarize the environment, providing additional disbalance of energy on its motion.

Addressing these problems, it was suggested that certain amino acid side groups – hydroxyls, carboxyls, imidazolyl, ammonium (from Lys) and guanidinium (from Arg) – are capable to participate in sequential proton transfer without ionization, i.e. without the net dissociation of a proton [142], [143]. It should be stressed, however, that when these side groups are ionized, they cannot be both a hydrogen bond donor and acceptor as required for sequential proton transfer. His^+^, Lys^+^ or Arg^+^ in protonated form have no lone pairs to accept a proton and deprotonated Asp^−^ and Glu^−^ are the hydrogen bond acceptors but have no proton to donate. By changing the ionization state of these residues, one can form or disrupt the H-bonding network.

Since this mechanism allows avoiding the participation of charge-carrying water molecules (performing protolysis or solvolysis [144]), the participating acidic and basic side chains of amino acid residues do not display conventional acid-base chemistry. They use the H-bonds to acquire a proton from an upstream proton carrier and then release it to a downstream carrier. Chemically, a lone pair of electrons on the oxygen atom acquires H^+^, while another pair releases H^+^. Throughout this process the hydroxyl remains as –OH, avoiding ionization to either –O^−^ or –OH_2_^+^, so that a chain of H-bonded hydroxyl groups could carry out the proton transfer over a considerable distance. This picture involving sequential proton hops from the donor to the acceptor is often referred as Grotthuss mechanism [145]. High proton conductance in neat water is also attributed to this mechanism [146].

The understanding how this process operates in terms of PT driving force extending at a long distance in dielectric medium is not satisfactory. The differences should exist in proton acidities of reaction participants at every step on proton pathway. In addition, the structural/energetic coupling of chain elements is a weak point in this description [134]. One can recollect the discussed above critical importance for PT of the H-bond length and angle that should exist for all elements of the chain. The translational irregularity and thermal fluctuations are always present in protein structures. Therefore, it seems unrealistic that the precise optimal alignment of H-bonds is persistent over a sequence of protein groups or trapped water molecules forming the relay. Polar residues Ser, Thr, Tyr, Glu, Asp, Gln, Asn, Lys, Arg, and His-may serve not only as potential chain elements but rather by providing the correct microenvironment for water molecules that actually conduct the protons [22], [147]. However, small water molecules in protein channels are not bound covalently and retain definite level of mobility [148]. The mechanism must exist that could allow the protein groups to act individually, without precise alignment of their energies and locations, but acting in proton relays in a synchronous manner.

The same question can be addressed to explain the light-activated double and triple PT reactions in organic dyes that are assisted by proton wires (Section 2.4). These wires connect the proton donating and accepting groups at distantly located well-defined positions. The strong advantages of these dyes as the models of biological PT are in the possibilities of performing the experiments with them in liquid solutions, in which one can vary the composition of wire-forming solvent molecules in broad ranges [108], [149]. In bulk neutral [83], [150] or aprotic [151] solvent these wires can be formed by protic guest molecules and by their various combinations possessing different proton-donating abilities [83]. It was observed that in wires formed by water, the PT proceeds with a very fast rate, whereas in alcohols the process is slower and is limited by the rate of wire formation. When the alcohol wire is formed in neutral solvent, the PT is also observed with a fast rate, suggesting its pre-existence. It was shown that if two different alcohol molecules form the H-bonded cyclic complex, they accelerate PT by accumulating their proton-donating abilities in an asymmetric concerted fashion [83]. Because the rate constant of ESPT was affected by the acidity rather than by the basicity of the alcohol, it was suggested that the rate-determining step here was the proton-acceptor action from a directly H-bonded alcohol molecule. Involvement of tunneling was indicated by a large H/D isotope effect [152].

Very important general conclusions can be derived from these results. The proton wires can be not only static but also transient; they can be formed and destroyed dynamically in solutions and often competing for H-bonds with bulk solvent molecules. This fact may be important for analysis of long-distance PT in proteins [153], in which the functional connectivity can be achieved not only by static structures (e.g. as in gramicidin and D channel in cytochrome c oxidase) but also transiently with highly mobile water molecules [22]. In these dynamic structures, it is easier to incorporate a mechanistic component of gating for performing the transporting or catalytic functions of proteins.

In order to describe collective behavior in H-bonded networks, the model based on Davydov's soliton theory was suggested [154] and resulted in further developments [155], [156]. This theory describes nonlinear solitary wave propagation in a network of H-bonded structures. Its experimental verification is pendant.

### 3.3 Protein molecules as reaction media for proton transfer catalysis

In different enzymes, the reaction sites are typically buried deeply within a protein matrix providing the channels for proton, electron and substrate/product transport between the active site and the surface of the protein. In enzyme catalytic cycles, the large-scale dynamics of molecular segments can be involved [157] and this fact can be witnessed as the strong solvent viscosity effects on catalytic rates [158], [159]. In contrast, if such dynamics is not involved, the enzyme redox reaction is sensitive to dielectric polarization of reaction site that can be observed as the reaction rate dependence of solvent dielectrics [160]. This means that the screening of reaction sites in protein matrix from highly dielectric water environment exists but it may not be fully efficient (or needed).

From the above discussion it can be understood that the preorganization of PT reaction site with optimal coupling of the donor and acceptor must be the general feature of any PT reaction in biological systems. A PT reaction will not proceed without precise reactive geometries between the enzyme, substrate, and reactive groups resulting in proper geometry in arrangement of donor and acceptor sites, their location at the shortest distance and their optimal H-bonding [161]. The structurally designed preorganization of local electric fields [162] that is known to produce dramatic influence on ESIPT, generating its Stark-effect modulation [92], can play a key role here by reducing the PT barrier heights. The experiments utilizing the vibrational Stark effect have provided convincing evidence supporting a major electrostatic contribution to enzymatic catalysis [161]. The role of amino acid residues located outside the active sites in regulating and enhancing enzyme activity can be observed by pH titration of ionizable groups and by providing the multitude of point mutations and demonstrating sometimes strong effects [163]. However, these effects are collective and their complexity makes the construction of electric field maps very challenging.

There are experimental evidences [38] supported by *in silico* modeling [164], [165] that even sub-Ångstrom length changes in the reactive complex can provide major effects on enzyme efficiency. The abnormally short-range H-bonds (<2.5 Å) are frequently observed in the active sites of enzymes [35]. Some authors attributed to these short hydrogen bonds their special type that is characterized by the equal sharing of a hydrogen atom between donor and acceptor [166]. In this case, the activation barrier for PT should be lower than the ground state vibrational levels of the reactant and product states (proton donor and acceptor), which may result in acceleration of enzyme reactions. However, in a series of elegant experiments on green fluorescent protein [167] and ketosteroid isomerase [168] it was shown that this may not be true – the H-bonds are short but protons are not delocalized.

Protein dynamics may result in non-productive or low productive conformations for catalysis, and the stabilization of unusual conformations or oxidation states have also been observed to control the reactivity in enzymes leading to distributed kinetics [169]. Such structural fluctuations can be revealed by different structural methods, including X-ray crystallography [170]. The electric field heterogeneity at the active sites is assessable by different methods, such as vibrational Stark spectroscopy and NMR, and the electrostatic perturbations can be induced by mutations or ligand binding [171]. Meantime, bioacatalytic reactions are commonly studied in ensemble-averaged mode [172] that does not allow demonstrating directly this type of heterogeneity. The distribution of small-scale interaction energies between aminoacid residues in proteins is clearly seen in site-selective Red-Edge effects [173], [174] in fluorescence of Trp [175] and different protein-bound fluorescent labels and probes [174]. In model systems, such selectivity can be demonstrated for electron transfer [176], [177] and excited-state energy transfer [177] reactions and also for proton transfer [26], [95], [178]. It was observed that the small-scale molecular dynamics leading to these distributions extends to much slower time scale (nanoseconds and longer) than the elementary ET-PT acts in enzyme reactions [26]. This fact was clearly witnessed in the recent studies of enzyme kinetics on the level of single molecules [179].

Thus, the stochastic thermal motions lead to conformational changes in enzymes and their ligands that result in configurations favorable for breaking and making of chemical bonds [10]. The involvement of these motions is thought to be much more important for PT than for ET due to its much sharper dependence on the distance, orientation and energetics of reactive groups, which was shown as the dispersion of fluorescence spectra for the protein-bound dyes [178]. Such dynamics has to generate temporally the most reactive configuration of the atoms involved, so that the PT donor and acceptor become transiently closer to facilitate the transfer.

The reorganization of electrostatic forces in catalysis by stochastic fluctuations or as the motions of pre-organized charges must have both electronic and nuclear contributions that evolve on different time scales. The response of electronic sub-system is the fastest component, and the slowest is the rotational and translational motions of charges and dipoles [86]. The ESIPT dyes covalently bound to protein molecules [102] or noncovalently attached to them [101] allow providing clear distinction between the effects of electronic and nuclear polarization and also of external H-bonding [91], [180]. Both in photochemical and biochemical reactions, the motions in electronic sub-system can be realized in a highly organized manner, in contrast to stochastic polarization effects that are commonly observed for reactions in solutions [181]. The highly organized protein structures, assembling both reactive and modulating groups in biochemical reactive sites [161], allow optimal exploration of the electronic and H-bonding abilities of reactants and ligands. The presence in the vicinity of the PT reactive sites of Trp-and Tyr-residues [175] may not be circumstantial, since the electronic polarization effect that they provide is the strongest.

Viewing at protein structures, biochemist must imagine quite a different picture than that given by high-resolution x-ray data. It should be the picture of electron, proton and radical pathways formed by intramolecular interactions [13], [182], [183]. If the localized active site is structurally resolved, it should be seen in the focus of electric fields formed by surrounding residues [161]. Such effect can explain the influence on reaction rates of distal mutations in protein structures [163], especially of those that change the charge. If the reaction site is distributed in space, the so performed electric field effect becomes also distributed, facilitating the choice of pathways for migration of electrons, protons and ions in channels [136], [184]. The role of water is much broader than being just a reaction medium. It is an active participant in PT channel conductance, in catalytic oxidation/reduction, as well as being a proton donor or acceptor to/from groups exposed on protein surfaces.

## 4 Ground-state enzyme catalyzed proton transfer reactions

Now we focus on PT reactions in enzymes, keeping in mind that they may be the elementary steps of complex chemical transformations that involve both reactant and enzyme. Coupling of PT with ET is quite common, and this coupling is diverse [44], being not only colinear but also orthogonal (along divergent pathways), such as in hydrogen producing [NiFe]-hydrogenases [185] and in *E. coli* ribonucleotide reductase [186]. The most typical examples of enzymes exploring PT as the basic mechanism are analyzed below.

### 4.1 Ketosteroid isomerase

**Fig. 17:**
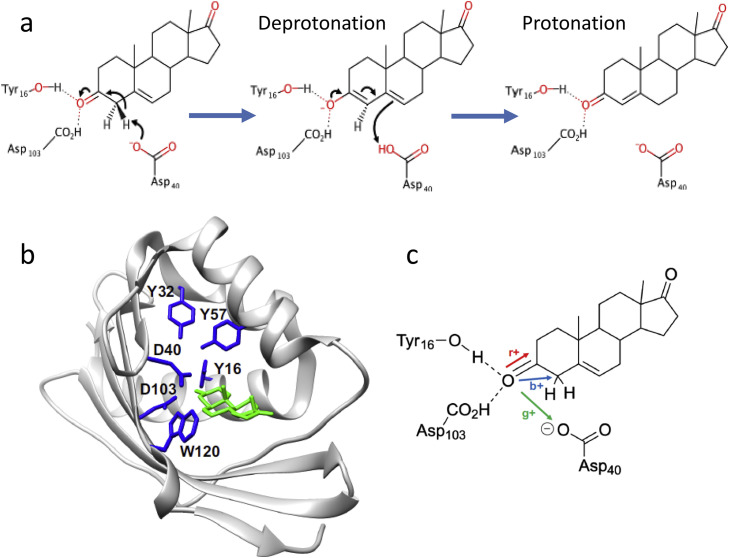
Ketosteroid isomerase (KSI) and its catalytic action [188]. (a) Steroid isomerization reaction catalyzed by KSI. (b) Full KSI protein structure (PDB code 1OH0) with docked steroid substrate. Residues included in the small-scale calculations are in blue color. The substrate is colored green. (c) The schematic presentation with red, blue, and green arrows in the directions of the external electric fields applied to the reaction site.

For our analysis of PT in enzymes, ketosteroid isomerase (KSI) is critically important. It is one of the most efficient enzymes exhibiting PT reaction as the key mechanism. For catalyzing the isomerization of steroid substrates it uses the same reaction path as in solution, but compared to the analogous reaction in solution it does that with a 10^11^-fold rate enhancement [187]. Its catalytic mechanism involves two sequential proton transfer steps (Fig. 17,a). One is the proton abstraction from the substrate to the enzyme forming a negatively charged intermediate, which is stabilized by the H-bonding network. Then the proton moves back to substrate but to its different position that is two carbons away, leading to a product with isomerized structure. Possessing one of the highest unimolecular rate constants, KSI does not demonstrate any change of protein conformation on binding the substrate, isomerizing it or subsequent product release. The strong H/D kinetic isotope effect observed for this reaction confirms that the PT is indeed its rate-limiting step.

Analysis of KSI structure allows identifying the network of short hydrogen bonds in its active site. The substrate is linked to Asp103 and Tyr16, extending to Tyr57 and Tyr32 (Fig. 17,b). The latter forming strong dipoles with their side groups coupled with H-bonds provide the focused electric field for the proper positioning of substrate and differential stabilization of the transition state (Fig. 17,c).

The ability to stabilize the KSI reaction intermediate through the active site H-bonding network was investigated with its mutant, D40N, which preserves the structure of the wild-type enzyme but allows mimicking the protonation state of residue 40 in the intermediate complex and in this way permitting experimental investigation of an intermediate-like state of the enzyme [189]. Without this complex, the side chains of residues Tyr16, Tyr32, and Tyr57 in D40N mutant form a hydrogen-bonded triad [190]. The close proximity of the oxygen atoms on the side chains of these Tyr-residues (∼ 2.6 Å) is remarkable and suggests the low-barrier proton migration. This network is thought to facilitate the deprotonation of Tyr57, leading to unusually high acidity of this residue (pKa = 5.8). Upon binding an inhibitor, an extended network of short hydrogen bonds is formed to incorporate the inhibitor and the residue Asp103. This network acts to stabilize a charged dienolate intermediate [168] and to focus the oxoanion electric field for significant increase of stabilization of transition state [15], [189]. The structural features of the oxyanion hole suggest that the H-bond formed to the reacting substrate is geometrically optimal in the transition state but not in the ground state. Thus, the strong H-bond acidity of the proton donor [191] and the unusually short distance between the donor and acceptor in the rigid active site are the characteristic features of this enzyme, leading to dramatic decrease of activation energy of the catalyzed reaction [34].

Thus, the catalytic mechanism of ketosteroid isomerase demonstrates clearly the role of electrostatic preorganization by fixing proximal charges and dipoles in biocatalytic proton transfer. Can this effect be modelled based on ESIPT reaction in synthetic dyes? Two types of model experiments can be recollected regarding this issue, though the molecular structures and experimental conditions were quite remote. In both cases, the designed 3-hydroxychromone derivatives were used. Synthetically, the perturbing charge was located at two opposite sites with respect to fluorophore with dramatic modulation of ESIPT reaction [92]. In other experiments, the dye was incorporated into the electrostatically anisotropic phospholipid membrane and the reversal in orientation of incorporated dye produced the electrostatic modulation effect also leading to its reversal [104]. These results suggest that in ESIPT systems, in addition to commonly observed dielectric reorganization of solvent molecules, the conditions for studying the preorganization more closely simulating the PT events in biocatalysis can be created.

### 4.2 Soybean lipoxygenase

**Fig. 18:**
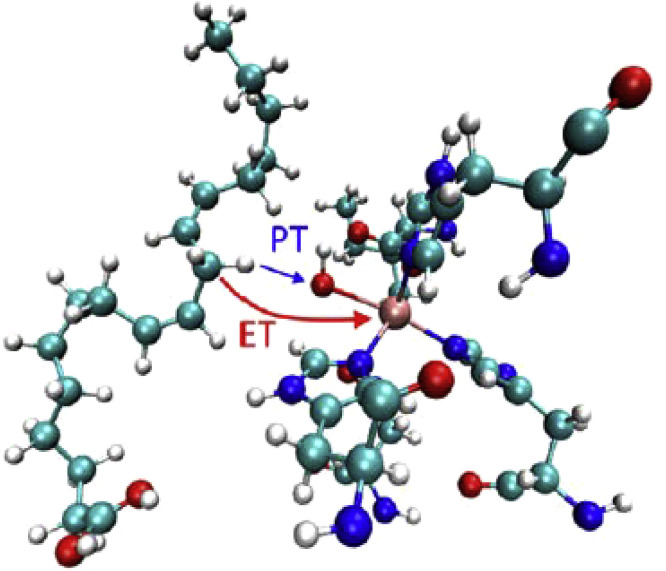
Schematic representation of the ET-PT reaction catalyzed by soybean lipoxygenase SLO-1 with the linoleic acid substrate [47]. The red arrow indicates the electron transfer from the π-backbone of the linoleic acid substrate to the iron of the cofactor, and the blue arrow indicates the proton transfer from C11 of the substrate to the iron-bound hydroxide form water. This ET-PT reaction is prototypical for hydrogen tunneling in enzymes.

The well-studied lipoxygenases catalyze the oxidation of unsaturated fatty acids. Being the targets for different drugs, they attract an interest of many researchers. Their numerous kinetic studies have been carried out using soybean lipoxygenase-1 (SLO-1) enzyme. SLO-1 is known for its extremely large H/D kinetic isotope effect (81) that demonstrates very weak dependence on the temperature, suggesting the involvement of proton tunneling in PT mechanism [125], [192]. The quantum mechanics (QM) calculations indicate that in the heart of catalyzed reaction is the proton transfer from the donor carbon to the oxygen acceptor that is coupled with the electron transfer from the π-system of linoleic acid to an orbital localized on the Fe(III) center [193]. Thus, a net hydrogen atom transfer is composed of ET and PT between distinct donors and acceptors (Fig. 18).

The proposed ET-PT concerted mechanism allows these reactions to proceed simultaneously without high activation barrier [125]. The reaction is exothermic by ∼5 kcal/mol, and the electron transfer is nonadiabatic due to weak coupling between the electron donor and acceptor. However, when this ET-PT reaction is compared with that occurring in photochemical models (See Section 2), it is retarded dramatically. This fact indicates the presence of some mechanism of gating that influences the rate, possibly by modulating the hydrogen transfer distance [125]. The recent QM/MM free energy simulations [40] point to a special role of protein dynamics in catalysis and suggest distinguishing between the role of overall protein environment (that is important for conformational sampling of active substrate configurations aligned for PT) from that involved in operation of the ET-PT reaction site. Primarily, local electrostatic effects influence this site. They facilitate conformational sampling of shorter proton donor-acceptor distances required for effective proton tunneling.

Based on the measured tunneling rate and using the calculated values of the electronic matrix element, the population of this tunneling-active conformation is found to lie in the range 10^−5^ − 10^−7^, and this fact can explain the so slow PT reaction rate [194]. Thus, we obtained a strong indication that there are not only ‘good vibrations’ but, mainly, rare structural fluctuations that are responsible for the gated retardation of PT reaction. These fluctuations making shorter the proton donor−acceptor distances generate a reactive configuration along the proton transfer coordinate, from which the reaction proceeds via nuclear tunneling [195].

### 4.3 Carbonic anhydrase

Human carbonic anhydrase II (HCA II) is one of the fastest known enzymes (the maximal turnover rate ∼10^6^/s), which utilizes a key PT step in its enzymatic reaction [196]. It catalyzes the following reversible hydration reaction of carbon dioxide:

CO_2_ + H_2_O ↔ HCO^−^ + H*^+^*.

Based on the structure with 0.9 Å resolution [41], the active site of HCA II was clearly identified as a cavity with a zinc ion coordinated in a tetrahedral geometry to three histidine residues (His94, −96, and −119) and a solvent ligand. The mechanism of catalysis comprises nucleophilic attack of the zinc-bound hydroxide on CO_2_, followed by PT from zinc-bound water via His64 to solution regenerating the active form. This latter PT step is thought to be the rate limiting at physiological conditions. The Zn atom and His64 are about 8 Å apart. A network of H-bonded water molecules connects them for providing the proton supply.

**Fig. 19:**
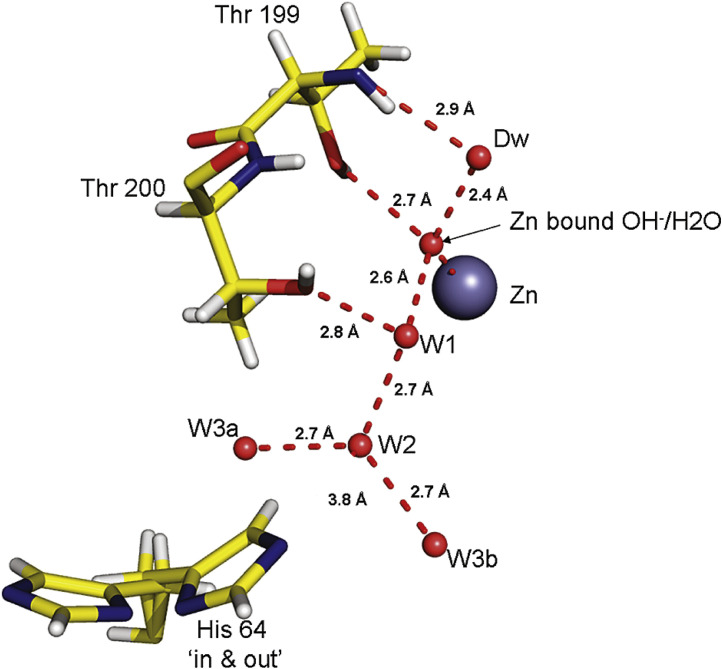
Ordered water network in the active site of human carbonic anhydrase HCA II [41]. A gray sphere represents the zinc atom. The oxygen atoms of water molecules (W1-W3) are depicted as smaller red spheres. Dotted lines are the presumed hydrogen bonds with the given heavy atom distances. Stick figures are the selected amino acids of the active site with shown both inward and outward orientations of His64.

The orientation of His64 can shuttle from inward (toward the active site) to outward (toward the solvent), suggesting its role in modulating PT and releasing proton in catalytic cycle. This role is similar to the gating observed in proton channels. The presence of titratable residue (His, Asp, or Glu) at the entrance to the proton channel enhances the proton conduction. A chain of water molecules mediates the multi-step PT during the catalysis (Fig. 19). This chain is immobilized within the structure, contacting with polar residues, and is stable against some of their mutations [197]. One water molecule, termed deep water, Dw, is very special. It is located in a hydrophobic pocket of the active site and forms an unusually short H-bond (the oxygen-oxygen distance of 2.45 Å) with the zinc-bound solvent molecule, suggesting the very strong bonding. It is expected to contribute to the low pK_a_ of the zinc-bound water and in this way to promote the proton transfer in catalysis.

### 4.4 Ribonucleotide reductases

**Fig. 20:**
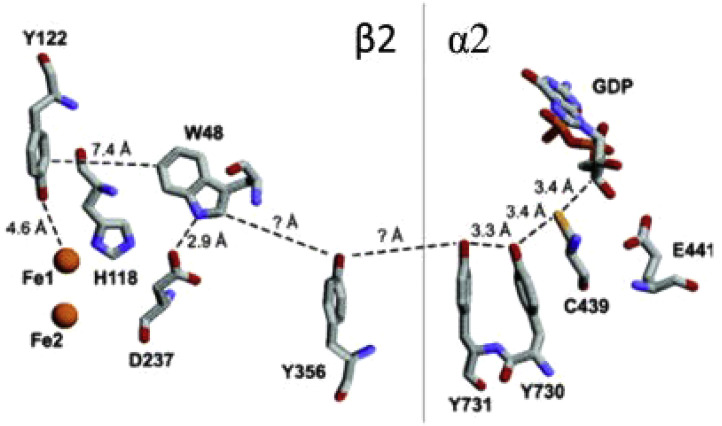
Putative ET-PT pathway for radical transport from Tyr122• to Cys439 in *E. coli* ribonucleotide reductase based on conserved residues, crystal structures of subunits β2 and α2, and a docking model [186]. Tyr356 has not been located in either the β2 or α2 crystal structure; other distances are taken from crystal structures.

Ribonucleotide reductases (RNRs) present an interesting example of enzyme, in which the multi-step ET-PT extends over a long distance, 35 Å [198]. In all organisms, RNRs catalyze the conversion of ribonucleotides to deoxyribonucleotides by removing the 2′‑hydroxy group of the ribose ring of nucleoside diphosphates. In these enzymes, the ET and PT steps are hidden in the kinetics by slow conformational changes. Based on protein structure and on the results of directed mutagenesis and pH titration, it was suggested that initiation of nucleotide diphosphate reduction requires a reversible oxidation by a tyrosyl radical located in subunit β of a cysteine in the active site of subunit α [199]. If being direct, such a long-distance ET would be low probable. Therefore, it was suggested that the radical transfer occurs as a multi-step process along a specific pathway involving a relay formed of redox-active tyrosines. A hopping mechanism involving the conserved amino acids Tyr122•→Trp48→Tyr356→Tyr731→Tyr730→Cys439 has been proposed (Fig. 20). Each step of ET is coupled to proton motion in the hoping chain, so that each oxidation necessitates a loss of proton coupled to a loss of electron [200]. A key role in radical transport is attributed to reversible electron transfer between Tyr122 and Trp48 [201].

Presented examples show clearly that the catalytic action of different enzyme classes requires some preorganization to facilitate and speed-up the reactions. Such preorganization can be dynamic along the given reaction coordinates [202]. A part of the reaction mechanism can be a long-distance proton transfer that requires for functional proton transport the multi-step tunneling, frequently called hopping. Highly resolved molecular structures demonstrate the presence of “proton channels” formed by polar amino acid residues alone or together with immobilized water molecules aligned in chains, and they are thought to participate in hopping.

Shorter donor-acceptor distances within the PT catalytic sites are seen in different enzyme structures. They allow recognizing the sites of catalytic proton transfer [27], [203], [204], [205]. This is in line with the results of photochemical studies demonstrating that a stronger H-bond accelerates the excited-state PT due to relative decrease in energy of the product state [17], see Section 2.5. Notably, the shifts in electronic charge density influence the H-bond distances, as it was shown for photoactive yellow protein [206], in line with the results of basic photochemical studies. All that makes mechanistic analysis of PT in proteins difficult, even in relatively simple cases. The responses to questions on proton motions stepwise or concerted [19], [207] and coupled or uncoupled with protein dynamics [141] are still unresolved to date.

The ESIPT models suggest new vision on the coupling of ET and PT and on the influence of protein and solvent environment on PT that should be accounted in the analysis of enzyme reactions. The actively discussed fundamental issues on proton tunneling, such as the role of barrier widths or heights [205], [208] or the involvement of “good” vibrations [209] can be resolved with the aid of ESIPT models. Strategic design of such models is highly needed.

## 5 Proton transfer steps in biochemical reactions activated by light

There is a great difference between the reactions activated by light and the thermally activated enzyme reactions discussed above. In most enzyme reactions, the kinetic studies demonstrate that the substrate binding and product release steps control turnover, indicating that in order to achieve perfectness, the fastest reaction steps were not under the evolutional pressure. In contrast, the light activation generates the short-living excited states, the energy of which has to be utilized in extremely fast way during the excited-state lifetime, before the excitation decay proceeds in emissive or non-emissive way. The most productive photochemistry is the ultrafast photochemistry that competes with all other pathways to release the excited-state energy [49]. This principle must operate in all events started by absorption of light quanta.

### 5.1 Flavoprotein light sensors

Photoreception is the property to absorb the quanta of visible light and to transform them into physiologically important signal. Light excitation of a pigment such as porphyrins and flavin adenine dinucleotide (FAD) bound to a photoreceptor protein lead to changes in the redox and protonation states that propagate to distal parts of the protein. The effectory signal changing protein conformation and molecular interactions starts a cascade of events, from transmembrane proton pumping to modulation of enzyme activities [210].

Among such signal converters is the family of incorporating FAD blue light receptors called BLUF [211]. In cells, these small proteins are coupled with many signaling proteins and provide to the latter the reversible activation. Their non-covalently bound flavin uses its isoalloxazine moiety as a blue light absorber. Since the whole process is initiated by light, the ultrashort (fs) light pulses can be used to start the reaction synchronously and to observe its development on a well-resolved fs-ps time scale. Upon the light absorption, the ultrafast (∼1 ps) light-induced ET-PT reaction between tyrosine residue and flavin proceeds with the involvement of glutamine and methionine residues yielding a neutral flavin semiquinone/tyrosyl radical. The subsequent rearrangement of hydrogen bonding networks that is key for signaling remains highly controversial [212], [213].

**Fig. 21:**
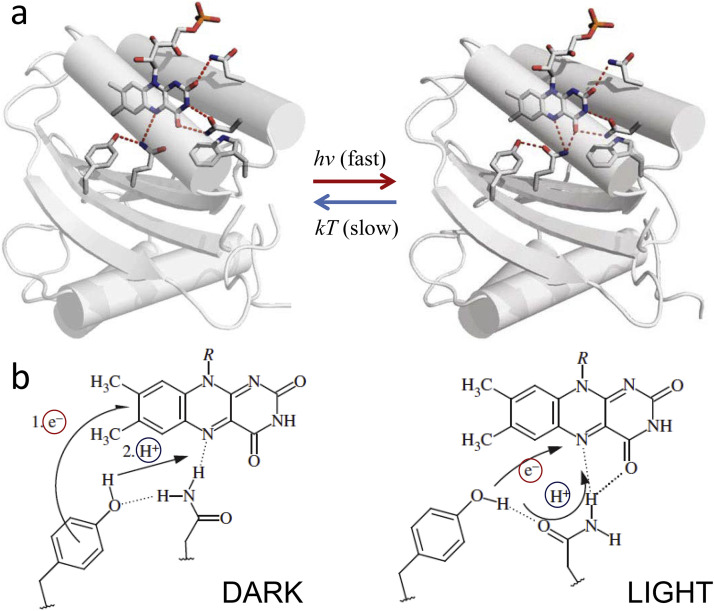
The images of BLUF domain of Slr1694 protein in the dark- and light-adapted states illustrating the putative glutamine rotation mechanism changing the H-bonding network [211]. (a) The H-bond network between the tyrosine, glutamine and flavin determines the light-induced proton-coupled electron transfer. (b) In the dark-adapted photocycle, the ET-PT reaction is sequential. In contrast, in the light-adapted state the neutral semiquinone intermediate is formed via concerted ET-PT reaction in about 1 ps.

The common feature of these photoreceptors is their presence in two functionally different light-adapted and dark states. Kinetic experiments show that in the dark-adapted state this reaction proceeds in a sequential fashion, where the electron transfer precedes proton transfer. In contrast, in the light-adapted state the same radical pair is formed by a concerted mechanism [214]. The natural explanation of this difference is in the H-bond switching between the flavin and its surrounding amino acid that preconfigures the system for proton transfer (Fig. 21).

The fact that the flavin-binding pocket of the BLUF domain undergoes a subtle rearrangement of the hydrogen network derives from comparison of the protein structures in the light-adapted and dark states (Fig. 21). Interestingly, the light-adapted state is formed during ∼100 ps and stays much, much longer [215]. The photoactivation reaction has to happen at a very fast rate, since the whole excited-state process has to be finished within the time window determined by sub-nanosecond excited-state decay. Also, the whole reaction cycle is reversible in a sense that at much longer times, within 10 ns, the photoexcited state falls back to the signaling state via the ground-state process with the recombination of bi-radical [216]. This downhill energy flow during the excited-state process triggers the events resulting in the appearance of chemical or structural memory, in which the extended conformational changes and intermolecular interactions take place. For being transmitted as an effectory signal, this memory should persist longer by orders of magnitude than the primary events [217], [218].

Thus, the photoreception is a multi-state process demonstrating broad distribution on the scale of time. Like in model excited-state reactions discussed above, the primary ET-PT events are very fast. Gating by making-breaking the H-bonds can be similar to that known in organic photochemistry, by changing the donor or acceptor conformation or their protonation states. The diphenyl benzazoles may serve as examples [219], [220]. The essential new feature is the long-term and reversible storage of the energy gained on electronic excitation in protein conformers. This raises a more general question, how the excitation to short-living fluorescent states can activate protein receptors and enzymes in such a way that they remain active at much longer times.

In addition to FAD-based receptors [23], these questions have to be addressed to the UVR8 photoreceptor using Trp-as primary light absorber [221] and photoactive yellow protein possessing *p*-coumaric acid chromophore [222], [223]. An analogy with the well-known photochromes [224] and metastable-state photoacids [225] may be useful for understanding these properties.

### 5.2 DNA photolyases

DNA photolyases use visible light and a fully reduced flavin cofactor FADH^−^ to repair major UV-induced lesions in DNA, so that the coupled ET-PT is in the heart of their action [226], [227]. By integrating fs-resolved spectroscopy and molecular biology techniques, the ultrafast enzyme dynamics could be analyzed in detail [228]. Particularly interesting is the so-called (6–4) photolyase that functions to repair the pyrimidine–pyrimidone (6–4) photoproducts. Its proposed catalytic photocycle for the repair of thymine (6–4) photoproduct includes the ET and PT reactions as the primary events [229].

The ET-induced proton transfer from the conserved His-residue in photolyase to the 6–4PP substrate is a key step in this repair photocycle, making the subsequent reactions kinetically irreversible. The successive elementary steps proceed naturally to an intramolecular PT from the hydroxyl group on the C5 of the 5′ base to the N3 nitrogen at the 3′ base to form a transient zwitterion. Then an oxygen atom attack on the C4 position at the 3′ base results in formation of a transient oxetane-type structure. The so complicated sequence of events occurs during sub-nanosecond times, demonstrating again the possibility of proceeding the ultrafast (fs-ps) ET-PT reactions in enzyme catalysis.

### 5.3 Photoinduced GFP transformations

There is a great interest to photophysics of green fluorescent protein (GFP) and its analogs in view of broad range of their applications in cell imaging and sensing technologies [230], [231]. The core GFP chromophore, 4-(4-hydroxybenzylidene)−1,2-dimethyl-1H-imidazol-5(4H)-one (*p*-HBDI), can be formed spontaneously in the cell by autocatalytic posttranslational cyclization and oxidation of a hexapeptide unit within a protein structure [232]. These proteins offer an example where the hydrogen bonding and proton transfer play essential roles in excited-state dynamics [233], [234]. The family of these proteins covers broad spectral range from visible to near-IR, and the PT dynamics is responsible to a significant extent for their spectroscopic properties including the generation of color. Here we concentrate on structural and temporal aspects of PT in the wild type protein (wtGFP) and its spectrally similar but brighter version GFPuv [235].

**Fig. 22:**
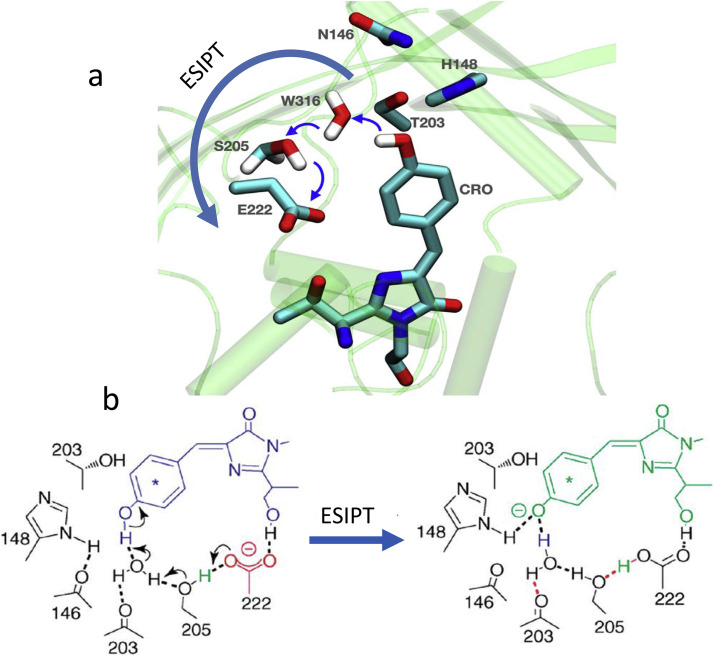
Prearrangement of residues (a) forming the proton transfer chain in wild-type green fluorescent protein (wtGFP) [239] and their chemical formulas (b) [240]. Proton wire formed in the protein structure results in energy absorbed at 370−400 nm in the A form (left) to be transformed to the excited-state proton transfer (ESPT) form I* (right). This stepwise ESIPT proceeds from the chromophore through the H-bonded network to Glu222 resulting in a very efficient anion fluorescence peaked at 508 nm. It arises from an anionic species formed in the excited state (I*) by deprotonation of the chromophore and concomitant protonation of the Glu222 carboxylate group. The direction of proton transfer from chromophore (CRO) to E222 via water (W316) and S205. The surrounding H148 and N146 help to anchor the active proton transfer path.

The proton transfer in GFP proceeds traveling at a large distance within the protein structure on a picosecond time scale. In a search for the proton pathway, a ‘proton-wire’ formed by the chromophore (the proton donor), water molecule W22, Ser205 and Glu222 (the acceptor) was found in the protein structure [236], [237]. The scheme of this multi-step process (Fig. 22) was suggested in early days [237] and was reproduced in many later publications [238]. In the ground state, GFP exists mostly in a protonated (neutral) form (A). Excitation with violet (370–400 nm) light generates the A* species, followed by fast intramolecular proton transfer, yielding the deprotonated (negatively charged) chromophore I*. The latter emits green light with a maximum of intensity at around 508 nm.

The application of spectrally resolved and time-resolved experiments have shown that ESIPT is not a single-step process and reveals the presence of intermediates [235], [236]. In any case, the whole process is ultrafast and proceeds on the time scale of 10^−13^–10^−11^ s, which is much faster than the emission rate of anionic form, demonstrating the lifetime up to ∼3 ns. It shows a pre-existence of a low-barrier or barrierless H-bond between the phenol group of the chromophore and the side chain of aspartate that ensures facile proton transfer.

The presence of a short (∼2.45 Å) H-bond (discussed in Section 2.1) is clearly observed in mutant S65T, H148D between Asp148 and the chromophore, which stimulated its focused studies [167]. Halogenated Tyr-residues were introduced into GFP to modify the chromophore acidity and to probe the effect of p*K*_a_ matching across a putative low-barrier hydrogen bond. Dramatic color-tuning of absorption spectra was observed on these substitutions, but even with the so short H-bond distance and near perfect affinity matching, no special effect of an ultra-short H-bond distance that may be thought to average the donor-acceptor proton location was found [167]. This means that the barrier to proton transfer exists and it exceeds the zero-point vibrational energy.

Natural and artificial mutations result not only in products of different colors. They offer an efficient tool to study the mechanisms of ESIPT in proteins of GFP family. Thus, in some of them the reaction rate was found to be retarded by several orders of magnitude. This fact was explained by conformational adaptation within the proton wire [241] or by a new proton pathway, in which the coupling water molecule does not occupy a stable position [242]. Such events may be present in other proton-relay systems formed of water molecules.

An importance of rigid environment stabilizing the H-bonding network is clearly seen from the attempts to design the small synthetic analog of GFP chromophore exhibiting the PT reaction. Primarily these attempts were not successful due to solvent-induced fluorescence quenching that are particularly strong in protic solvents [243], and only the compound that folds into the form stabilized by intramolecular H-bond [244] allowed providing spectroscopic and kinetic studies. In contrast to the native *p*-HBDI, the new compound *o*-HBDI possesses an H-bond that closes the seven-membered-ring and thus allows observing the excited-state transformation as an intrinsic proton-transfer reaction. The o-HBDI tautomer emission demonstrates oscillations on ∼100 ps time scale. Frequency analysis derived from providing the ultra-short pulse gives two low-frequency vibrations at 115 and 236 cm^−1^ that correspond to skeletal deformational motions associated with the hydrogen bond. The authors conclude that the ESIPT in *o*-HBDI is triggered by low-frequency motions and may be barrierless along the reaction coordinate. This conclusion was confirmed in the studies of *o*-HBDI analogs [245].

Linking photochemistry and biochemistry, the proteins with different light-absorbing centers have evolved to detect light and harness photon energy to bring about downstream chemical and biological output responses, starting enzyme reaction or producing the emission of light. The coupling of ultrafast ET-PT steps with much slower steps, such as conformational propagation together with rearrangement of H-bonding networks, is their important feature. They proceed along the sequence of intermediates that are very hard to resolve. Recently developed time-resolved serial femtosecond crystallography (SFX) technique, enabling direct visualization of ultra-fast changes in light-activated proteins can be of use to address these issues [246].

## 6 Understanding the operation of transmembrane proton channels

Providing and controlling proton transport across biological membranes is one of the most fundamental but also the most complicated properties of living systems. In such reactions, the movement of proton occurs over significant distances, and this is not possible in one-step. The process should necessarily involve many elementary PT steps along the ‘proton wires’ and imply a significant structural organization of its path. Different structural, energetic and kinetic constraints have to be satisfied for making this reaction specific and efficient. Proton entry and release at the channel terminals can determine the observed kinetics. Meantime the motion within the channel remains in dark, with no characteristic emission detected. Therefore, the implication of simple models is very difficult here, and this explains the fact that many important issues are still unresolved.

### 6.1 Proton wires in membrane protein structures

Despite many efforts of researchers, several key questions are still the subject of some controversy. The first question, what is actually transferred across the membrane. Because proton (H^+^) is a very small charged particle, its electrostatic binding energy is very large, and in condensed phases, it does not exist independently. In aqueous solutions, it is always present in complexes with water molecules. Such complexes are commonly presented as H(H_2_O)*_n_*^+^ [247], so that the smallest of them, H_3_O^+^ (hydronium) and H_5_O_2_^+^, are considered to be rather stable, and it is their diffusion observed in bulk water [248]. One may consider that hydronium is similar in size and solvation characteristics to Na^+^ ion, so its low selectivity may be expected in channels, but this does not happen! Keeping in mind that water channels (aquaporins) are impermeable to protons [249], we have to assume that the long-range H^+^ transfer proceeds in the ‘naked’ form, without any carrier, in a channel that is selective for proton only.

The second question, if the proton channels as the H-bond connected wires really exist and if yes, how they can be identified in protein structures. This question was raised many years ago and it stimulated discussion of possible solutions [143], but still at present we have only partial answer. Tentatively the wires can be ascribed to organized chains of water molecules seen in high-resolution X-ray structures [197], [250], [251] and by testing the effect of disruption of these chains in genetic mutants [252], [253]. Oxygen, nitrogen and sulfur atoms of the amino acid residues together with oxygen atoms of the trapped water molecules linked with H-bonds can form these wires inside proteins. Their identification is not easy [22].

The third question can be raised about the basic mechanism of proton motion over significant distances, often of the order of a membrane thickness. This motion necessarily involves many elementary proton-transfer steps and implies a significant both structural and dynamic component. In bulk water, the stepwise hopping from one protonated form to the other (between H_3_O^+^ and H_5_O_2_^+^) is commonly described based on the so-called Grotthuss mechanism postulating repeated cleavage and re-formation of hydrogen bonds [145], [254]. The OH groups of water molecules possess a property to be both H-bond donors and acceptors and therefore the short proton-transporting wires are occasionally formed even in bulk water, as we have seen in solvent-assisted ESIPT in organic dyes (see Section 2.4). However, it is still unclear if proton translocation along preformed one-dimensional chains of H-bonds strictly follows the Grotthuss mechanism. The key difference between the water bulk and unidimensional proton wires is that in the latter case the PT is facilitated by a favorable pre-alignment of water molecules, whereas in bulk liquid water the significant solvent reorganization may be required prior to PT.

**Fig. 23:**
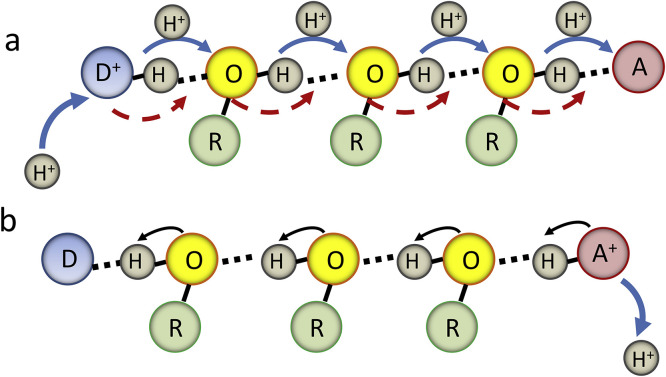
The scheme illustrating the possible operation of proton wire formed of the H-bonded chain of amino acid side groups and/or of trapped water molecules according to “hop-and-turn” Grotthuss mechanism. (a) Initial steps. Proton (H^+^) is taken up from the donor (D) and enters the chain formed by OH-groups of H-bonded oxygen atoms (O) serving as both donors and acceptors. It propagates through the chain in a stepwise manner in ‘accepting proton’ positions (blue arrows) coupled with the changes of electronic polarization (red dashed arrows). (b) Final steps. Proton motion changes the H-bond configuration in the chain to ‘donating proton’ positions. When proton reaches the final acceptor (A^+^), it is released to the medium and the configuration of H-bonded chain returns to initial position (black arrows). The symbol (R) refers to the second hydrogen atom of water or to the remainder of the amino acid side chains leading to the protein backbone.

The proton wire should be a composition of the proton donor at its one end and of the acceptor at the opposite end connected by chain of groups of atoms serving simultaneously as donors and acceptors (see Fig. 23). In the Grotthuss ‘proton exchange’ mechanism no proton moves more than one bond, as at each step the proton acceptor takes proton from the neighboring proton donor and exchanges it for another proton. The moving element here is the proton charge rather than the actual proton, so a proton would attach itself at one end of the wire, and emerge at the other end as a different proton. Overall, the coupled transfers lead to a proton rapidly leaving the input side and appearing at the end of the chain. However, at each step the H^+^ transfer requires the solvent reorganization around the proton-receiving species (pre-solvation) to develop a coordination pattern for hopping to the acceptor next in sequence. This reorients the hydrogen bond connectivity along the chain.

After the proton transfer step, the H-bonding network of the whole chain should be restored to complete the translocation cycle and for accepting the next proton in sequence. Thus, in order for the next proton to transfer in the same direction, the hydrogen bonded chain needs to fully reorient to its initial state [142]. The stable continuous H-bonded structures should be arranged either as depicted in case (a) or as in case (b) of Fig. 23. Protons must travel one by one, since the presence of several protons in channel should break this connectivity and screen the transmembrane electrochemical gradient, the driving force of this reaction.

High cooperativity between molecules forming the proton wire should exist [255]. The return to initial state of the wire for accepting the sequential proton may take time and increase the length of each step to 1–2 ps, even if there are no branching and returns [256]. Also, the change in electronic polarization that accompanies this multi-step process may be strongly involved [257].

Achieving full understanding of proton channel operation on a basic level is a difficult task. It is evident that the transfer of multiple protons in such hydrogen-bonded networks occurs one proton at a time. In a series of recent experiments [258], [259], a model was constructed, in which the proton transport occurs from initial phenolic proton donor to the pyridine-based terminal proton acceptor by a Grotthuss-type proton wire made up of a chain of benzimidazoles that form a hydrogen-bonded network. It was interesting to see that an increase of number of bridging benzimidazoles leads to decrease in efficiency of oxidation, and the substitution of the benzimidazoles with electron-withdrawing groups (e.g., trifluoromethyl groups) allows maintaining it on a high level.

What is lacking is the proper description of proton transfer in channels as a multistep process occurring in structurally heterogeneous media, since those are the protein interiors. On the pathway, different donors/acceptors may possess different proton affinities. Therefore, for efficient proton transfer, the energetics at every step has to be correlated with the energetics of other steps. This can be realized in several ways by optimization of donor-acceptor distances and of electronic polarizations along every PT step. The proper location of polarizable groups and the proper application of local electric fields are needed for that.

### 6.2 Conductivity and selectivity in proton channels

The living cell is impermeable to protons. Moreover, for maintaining the pH balance and formation of electrochemical gradients, protons should be moved out from the cell in a controlled manner, and proton channels perform this function. Proton transfer through the membranes is different from the transfer of metal cations, such as Na^+^, K^+^ or Ca^++^. The ‘proton exchange’ mechanism depicted above explains easily the selectivity of proton channels over the channels for other cations. For achieving specificity, the metal cations use special elements of channel structures called the selectivity filters that provide control by both steric (size) and electrostatic (charge) effects. In contrast, every elementary act of proton motion in the channel is specific to proton only and cannot be performed by any other ion [130]. Moreover, any additional space that is required for motion of metal cations does not exist in proton channels, and also the electrostatic field and electronic polarization effects that are quite important in both cases provide different barriers to charge motion [260]. Water wires are thought to be the most efficient elements for providing the proton current [261].

Interesting in this respect is the analysis of operation of gramicidin channel that is able to conduct both protons and alkali cations [262]. It has a pore lined up by about seven water molecules. It was found that the effective diffusion coefficient for H^+^ in this channel is at least 20–100 times higher than for any other ionic species or for the net flux of water [263]. This suggests that the mechanism of so high proton conductance is different; the proton motion proceeds independently of molecular or ion diffusion. This result is in line with a Grotthuss-type mechanism. The postulated reorganization of H-bonds suggests that such reorganization (depicted in Fig. 23) may be the rate-limiting factor in the process of proton conduction.

The electrostatic polarization [257] and permanent electric field formed by protein dipoles adapted to the transfer of charge must also be important. Interesting is the role of four essential Trp-residues in this channel [264]. They are known as strongly electronically polarizable dipolar amino acids, and their dipolar interactions probably compensate the energy cost of proton desolvation [265]. The other factor modulating the proton conductance can be the dipole potential of the membrane [104], into which gramicidin is incorporated. It was shown that the conductance for proton and for alkali metal cations changes with variation of dipole potential in an opposite manner, so that its increase enhances the conductance of protons but reduces the conductance of these cations [266].

New ideas appear when the behavior of efficient proton channels is compared with that of aquaporins, the channels that are intended to transfer water molecules through cell membranes at high rates. Surprisingly, their proton permeability is extremely low [267]. The water dynamics in this channel is very fast, and transient formation of wires is quite possible. So what specific design features prevent the conduction of protons? It must be the high electrostatic barrier and low level of polarization effects that could potentially reduce this barrier. The calculations [268] suggest that here the limiting factor is a positive electrical potential near the center of the channel generating the barrier height for a proton on the order of 25 kcal/mol. In the absence of a specific repulsive electrostatic interaction, the barrier appears due to the loss of solvation energy leading to insufficient electrostatic stabilization of proton when passing through the channel.

Thus, the ‘proton-selective filter’ can be any proton-dissociating amino acid residue on a conduction pathway of the proton channel just because such a group can bind protons but no other cations! The fact that proton conduction is gated by the mechanism that is specific for protons but does not need any special arrangement of structures is supported by the early data on synthetic peptides [269]. When incorporated into phospholipid membranes, they demonstrate manifold greater permeability for H^+^ than for any alkali metal cation at similarly high concentrations. However, selective filters may also be involved, such as charged Asp^−^ residue or Asp-Arg-linkage attributed to hH_v_1 channel [270].

Summarizing, the transmembrane conductance of protons operating in membrane channels or pores is different from that of metal cations or water. They are selectively activated, blocked and their conductance regulated by quite different means. Important is keeping the connectivity of H-bonding networks that can be transiently disrupted by thermal fluctuations but protected by side groups in the structure.

### 6.3 Driving force of proton transfer in channels and its gating

The voltage-gated proton channels (H_V_) are the proteins that are able to mediate the rapid movement of protons across the cell membranes at very low physiological concentrations (∼10^−7^ M) and with the rates that are much higher than that for other ions [271]. Regulating ΔpH between two sides of the membrane, H_v_ adopts two functional states, open and closed. In open state, each channel allows H^+^ to cross the membrane, moving along the electrochemical gradient with the rate up to 10^5^ protons per second. The chemical part of this gradient reflects the concentration difference across the membrane. Moving from high to low concentration, the H^+^ ions provide outward current, decreasing the cell acidity. The ΔpH values influencing the difference in proton affinities between its donors and acceptors on two sides of the membrane (ΔpK_i_) cannot be very large [272], and the other component of the driving force, the membrane potential, is thought to be more effective. However, it is not monotonous across the membrane, and, moreover, it is composed of dipole, surface and transmembrane potential contributions that are of different values and even of different sign [273]. It produces the same effect on the ions of the same charge, but because of unique mechanism of proton conductance, the H_v_ channels are so selective.

The ‘voltage-gating’ is the switching between the channel open and closed states operating in response to cellular changes of membrane potential. Most voltage-gated ion channels open up when the cell membrane is depolarized. But there is no satisfactory explanation of the proton channels gating response to ΔpH [274]. The opening/closing of the gate is modulated by pH change both inside and outside the cell [275], so that titratable side chains of particular amino acids with their pK_a_ within the operational pH range may be involved. However, the presence of proton-dissociating groups at both channel terminals is not sufficient to provide such effect of gating [130], [272]. The conformational switch between water-oriented and protein-oriented protic group may result in a “conformationally controlled pK‐switching” [276] serving as the gate in proton channel.

**Fig. 24:**
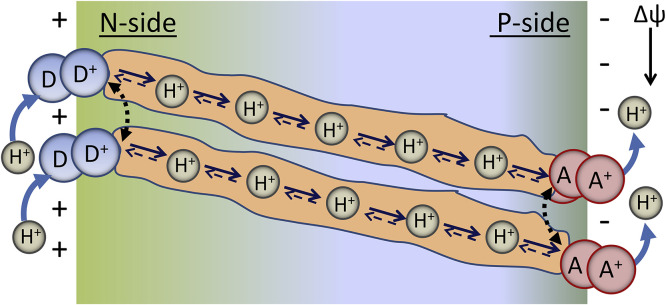
The scheme illustrating the operation of dimeric proton channel. The channel transfers protons (H^+^) across the membrane from inner cell volume (N-side) to cell outside (P-side). The proton flow decreases the acidity appearing as a result of intracellular metabolic reactions. The unidirectional proton flow is driven by transmembrane electrochemical gradient (Δψ) that tends to decrease (+ and - signs depict the difference in proton occupancy). PT starts with proton capture and deprotonation of the donor (D) and ends at the acceptor (A) providing proton absorption from the channel and then its release to the medium. The pH sensors composed of pH titratable groups are located at both terminals of the channels. Their cooperation in dimers is shown with black dotted arrows.

The other mystery is the operation of proton channels as dimers in mammals and many other species, since each monomer has its own conduction pathway and can function independently; but the dimer demonstrates the gating cooperativity [130]. Allosteric effects are thought to be involved here [277]. The schematic presentation of such dimeric hH_v_1 proton channel is given in Fig. 24.

## 7 Proton pumps in photosynthesis and cellular respiration

A variety of means is used by biological systems to generate the energy by providing proton transfer in thermodynamically uphill processes with the result of forming its transmembrane gradients. Bacteriorhodopsin represents the simplest system that allows formation of such gradient. In green plant photosystem II (PSII), its generation is coupled with the production of oxygen in reactions that use the energy of light to split water. In oxidative cell respiration, the chemical energy also comes from transmembrane proton gradient on mitochondrial membrane, but in this case, the protein cytochrome c oxidase (CcO) uses molecular oxygen as an energy source generating water in an exergonic process. Despite different sources of energy (visible light absorption or oxidation of the substrates), the proton-motive force provides the transfer of protons and electrons across the membrane. Here the cases of BR, PSII and CcO are discussed without going deep into structural details. Their common feature is the presence of proton loading sites (PLS), the mechanism of operation of which is obscure [239] and will be discussed here with the formulation of simple ‘proton lift’ concept.

### 7.1 Bacteriorhodopsin

Bacteriorhodopsin (bR) is the membrane protein that was found in purple bacteria. It has seven transmembrane helices and a central retinal chromophore. Demonstrating the most fundamental type of photosynthesis, it operates as the direct light-driven uphill proton pump, [22]. Its reaction cycle consists of proton transport from the cell interior (N-side) to the cell outside (P-side), transforming the energy of light quanta into transmembrane proton gradient [278]. The stored energy is used in biosynthetic processes.

**Fig. 25:**
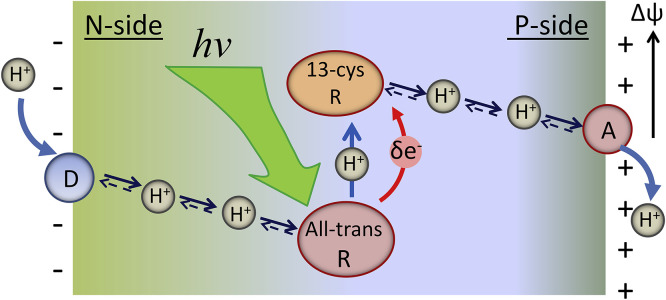
The simplified scheme explaining the motion of proton against its transmembrane gradient in a photogenerated proton pump bacteriorhodopsin. Reaction starts with the absorption of green light by proton-loaded all-*trans* retinal that upon isomerization to 13-*cys* retinal switches from proton acceptor to donor, lifting the proton energy. The two sections of proton channel are involved in this reaction. The section at the cytoplasmic negative N-side serves for supply of protons to the ground-state retinal-Schiff base. In the excited state, proton is supplied to extracellular section of the channel (positive, P-side), which leads to its transfer and release to outer space. The free energy (Δψ) becomes higher than that at the N-side, creating the proton gradient.

Retinal is located in the middle of protein interior covalently bound via protonated Schiff base to Lys216 residue. Forming the so-called proton location site (PLS), it is coupled with two sections of proton wires, from interior and from extracellular sides (Fig. 25). As in other studied cases of proton channels, they are the ordered chains of proton carriers formed of water molecules [147] and amino acid residues [222]. The high-resolution bR molecular structure [279] allows to tentatively assign the location of these wires, and the mutational studies [280] ascribe to them the gating function. The mechanism of gating that controls the access for protons of the protein interior from or to the outside is probably the same as in proton channels discussed in Section 6. The gate should ensure unidirectional proton flow, blocking the reverse transfer. However, there is no continuous proton wire throughout the pump molecule, as a central light absorber and proton acceptor/donor causes its disruption into two halves. Its function is to supply energy for proton motion against electrochemical gradient.

The net reaction scheme in this case is very simple. One photon of visible light generates the transfer of one proton from inner to outer side of cell membrane:

H^+^(inside) + *hν*(max 568 nm) → H^+^(outside).

In reality, it is a complicated process of the well-controlled multi-step unidirectional proton flow against its concentration gradient, the complete understanding of which is lacking [281], [282]. The primary event involves the reversible isomerization of the all-*trans* retinal to a highly twisted 13-*cis*, 15-*anti* configuration. The role of proton loading site (PLS) that modulates proton uptake and release is assigned to this site together with neighboring residues, the operation of which requires the coupling of photoinduced change of retinal conformation with proton current [239].

Retinal has a polyene backbone with long conjugated double bonds, and its isomerization is an ultrafast process. Ultrafast time-resolved spectroscopy allowed detecting a series of sequentially generated intermediates (I, J, K, L, M, N, O) in a reaction cycle. The first intermediate generated after isomerization of retinal to the 13-cis form is the K intermediate [283]. It forms in 3 ps; it is accompanied by change in electronic polarization [284] but produces only minor changes in conformation of surrounding groups. Deprotonation of the Schiff base of 13-cis retinal and its coupling with protonation of Asp85 during the subsequent L→M transition ultimately results in proton translocation to the extracellular side of the membrane.

The driving force for proton transfer in both sections of a channel comes from fitting their elements to proton affinity gradients. A key reaction, however, is the vectorial uphill (in energy) proton motion that requires the acquisition of so high energy level that must be sufficient for the transfer against electrochemical gradient existing across the membrane. Therefore, it is required that the photoinduced deprotonation and reprotonation of the Schiff base should be coupled with the reversible switch between proton binding from the downhill side open to the cell interior and its release at the membrane uphill part of the channel that is open to the cell outside (see Fig. 25).

This “proton lifting”, the jump of proton energy that accompanies the photoisomerization of retinal and estimated to be ∼50 kJ/mol [285], is probably based on the same mechanism as that of “super photoacids” known in organic photochemistry [286], [287]. These compounds possess strong proton affinity in the ground state but dissociate it in the excited state, demonstrating dramatic drop of pK. This occurs due to transfer of electronic charge in the excited state making the PT donors much stronger donors. In photoacids, this process is reversible leading to rapid re-protonation on return to the ground state. Nevertheless, if the dissociating and associating is the proton coming from the same site and returning to it, the energy should dissipate and no pump action could occur. This should not happen if the transfer occurs not to solvent but to closely located proton acceptor [288], [289]. Instead of ground-state back association with the donor, proton can be loaded to proton channel leading to cell outside.

Following this mechanism, the Schiff base in the ground state must operate as a strong proton acceptor terminating the inner segment of proton channel (the N side). Upon excitation and isomerization of retinal, the Schiff base proton acceptor is switched to become the high-energy proton donor. It can maintain only for a very short (ps-ns) lifetime of the excited-state *cys* isomer. Therefore, the postulated PT reaction from the protonated Schiff base to the H-bonded Asp85 at the outer segment (P side) should be also fast conforming to the time window given by the retinal excited-state lifetime. Thus, the rise of pK of Asp85 should be simultaneous with the retinal isomerization to enable it to serve as an excited-state proton acceptor.

The next steps in proton transport are slower, allowing the conformational changes to occur for preventing the back reactions. These changes are detected by time-resolved techniques on µs-ms time scale [290], [291], [292]. Their presence was established in high-resolution crystal structures studying the illuminated and ‘dark’ protein forms and at different pH values [293], [294], [295]. Since the changes in retinal-Schiff base electronic sub-systems generate strong redistribution of charge, the changes in electrostatic interactions trigger these conformational changes. The overall process is slow, since it should be time-limited by proton release at extracellular side. This rate is probably correlated with the ground-state *cys*-*trans* isomerization (closing the photochemical cycle) and the capture of the next proton from the cytoplasmic side via Asp-96 residue serving as primary proton donor.

### 7.2 Photosystem II

Electron and proton transfer reactions are at the heart of photosynthesis. Photosystem II (PSII) is a huge multi-subunit protein complex located in the thylakoid membranes of green plants and cyanobacteria. Harvesting sunlight, it transforms solar energy into strong transmembrane proton gradient that provides energy to biosynthetic reactions. In addition, PSII is the only known enzyme capable of oxidizing water with the generation of oxygen, acting as a light-driven water:plastoquinone-oxidoreductase [296], [297]. Thus, PSII utilizes the sun's energy to split water into protons, electrons, and oxygen, producing the basic reaction of transmembrane charge separation:

2H_2_O + 4*hν* (red) + 2PQ + 4H^+^(stroma) → 4H^+^(lumen) + 2PQH_2_ + O_2_↑.

The overall process comprises three types of reaction sequences: (1) photon absorption and excited-state energy trapping by charge separation, leading to the ion radical pair formation, (2) oxidative water splitting into four protons and molecular dioxygen at the water oxidizing complex, and (3) reduction of plastoquinone (PQ) to plastoquinol (PQH_2_) [296]. The whole series of these events in time and energy domains is not understood in full, and we will discuss only the particular case of realizing the mechanism of electron-proton coupling.

At the first sight, this process resembles the ET-PT reaction in organic dyes, where the translocation of four coupled *e*^−^/H^+^ pairs occurs by absorption of four quanta from common donors to different acceptors (see Fig. 2,d). But closer look reveals that the difference is dramatic [298]. Here the reaction is extended to a much larger length scale, of the width of biological membrane (∼50 Å). This distance is too large for one-step tunneling, not only of proton but also of electron. It is also known that at sufficiently large distances (∼10 Å and longer) the multistep ET is much more efficient than a single-step hop [299]. A number of pigments and the side groups of amino acid residues must be present as the basic elements to mediate the electron and proton transfer reactions.

Central in these events is transforming the energy of light absorbed at the site that is a special assembly of two chlorophyll molecules, known as P680. The function of proton loading site (PLS) is attributed to this locus [239]. Absorbing the light quanta mediated by large chlorophyll antennae, the P680 site in electronically excited state performs the charge separation leading to cascade of events of electron transfer. In order to capture the electrons in the catalysis of water splitting, a high input of energy is needed. It is taken from the excited P680 when it relaxes to the ground state. In this process, the excited P680 generates the high-energy electron-deficient state (hole) that operates as an extremely efficient electron acceptor [300]. This conversion provides energy to the whole process.

**Fig. 26:**
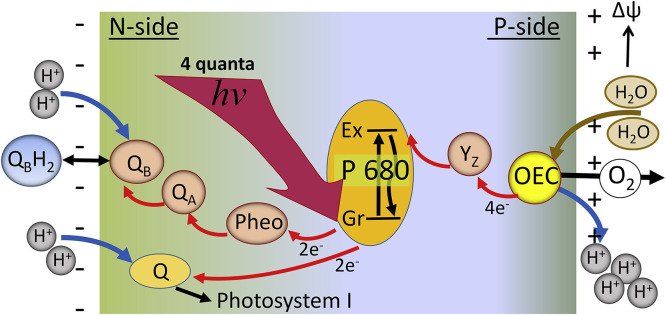
Schematic presentation of the PSII structure illustrating the sequence of ET-PT events that occur on absorption of light quanta by coupled chlorophylls P680. The capture of four electrons by excited P680 via a redox-active tyrosine (Y_Z_) starts the coupled events at oxygen-evolving complex (OEC) generating the consumption of two water molecules with the release of molecular oxygen to gas phase and of four protons to cell exterior generating the membrane potential. On relaxing to the ground state, P680 releases two electrons, so the negative charge proceeds on the N-side in a sequential way via pheophytin (Pheo) and quinone Q_A_ to Q_B_ yielding with binding two protons the reduced plastoquinone (Q_B_H_2_). The latter translocate to solvent being exchanged for Q_B_. The other quinone by accepting two electrons is able to bind two protons and lead to Photosystem I. The release of four protons at biomembrane P-side creates the free energy (Δψ) gradient.

The chemical transformation proceeds in Mn_4_CaO_5_ cluster, which is the heart of oxygen evolving complex (OEC) located at a distance from central P680 chlorophylls, close to the lumen [301]. This reaction starts from abstracting the electrons from the water-splitting site at the extracellular lumen side of cell membrane (P-side). Here two water molecules are split into hydrogen ions (H^+^) and oxygen atoms and the latter combine to form molecular oxygen (O_2_), which is released into atmosphere. The four protons liberated in this reaction translocate to the membrane surface. They form transmembrane proton gradient providing the storage of energy and its use in chemical synthesis (Fig. 26).

An efficient ET relay should couple these two processes. According to structural data, the redox-active Tyr-residue Y_Z_ is involved in this relay [111], [298]. Notably, the chemistry reaction step proceeds much slower than the excited state lifetime of P680 (ns) and the rate of tunneling (hopping) in the electron relay connecting P680 and the reaction site, OEC. In this water-oxidizing reaction, the intermediates with sequentially increased lifetimes from 40 µs to 1.6 ms have been identified (the so-called S_0_-S_4_ cycle) [302]. They allow accumulating in redox states sufficient energy for splitting of water. Four electrons and four protons are released sequentially one-by-one and with different rates at different steps during this cycle [303].

As any organic photochemical system, the P680 site should respond in one photon – one electron manner. Therefore, the highly energetic oxidation equivalents should be temporally stored in order to connect this one-electron process of electronic excitation to the four-electron process of forming dioxygen. Thus, the problem of coupling the events occurring with different number of electrons and on different time scales is actual here, and different gating mechanisms should be present; their origin is debated [304], [305].

Relaxing to the ground electronic state, the P680 complex must demonstrate dramatic transformation of its properties, shifting from the electron acceptor to the electron donor. Its donating ability is needed for making the electron capture irreversible, for supplying the energy to the whole system and for preparing P680 for the next light absorption step. The electrons are released to sequential relay of pigments that transport them to cytoplasmic N-side (strom) [304]. Here they are used to activate (reduce) an electron molecular acceptor quinone Q_B_ making it a strong proton acceptor. When it is in the resting oxidized state, the pK_a_ is low enough (pK_a,ox_ < pH) to capture protons. Accepting electron, its proton affinity becomes sufficiently high for binding protons from the solution (pK_a,red_ > pH). Such types of coupled ET-PT reactions are well-known in photochemistry [16]. In this particular case, the quinone can bind two protons becoming Q_B_H_2_ (see Fig. 26) and then exchange with unbound quinone in extracellular phase on a ms time scale [306].

In the protein structure, the channels for the substrate water supply and for the product protons release at the P-side were identified [307], but some questions remain [308]. The bulk water, to which the water channel is exposed [184], makes the supply of water molecules to the reaction site thermodynamically favorable. Meantime, the protons are released to a phase possessing high proton density. The driving force for this process, its energetics and kinetics, is yet to be elucidated.

Another important issue is how the electron and proton motions are coupled with each other at each elementary step of PSII reaction. Since they have to move in opposite directions (at the N-side: electrons to P680, to the inside of membrane, and protons to outside), the familiar to photochemists principle of avoiding the high energy barriers by their coupled motion (see Section 2) does not operate here. Fig. 26 allows explaining how Nature has found the way to avoid this problem. Protons do not move the long way across the membrane. The protonation states of chlorophylls forming P680 do not change their protonation states and there are no proton channels leading to them. Protons are just exchanged at membrane surfaces at both membrane sides providing the change of protonation of quinones and Mn_4_CaO_5_ OEC [297]. The electron transfer only with the supply of excitation energy through the reaction cycle allows formation of transmembrane proton gradient.

Thus, in Photosystem II the light-driven electron transport generates the transmembrane proton gradient. Importantly, the electrons travel across the membrane without actual transfer of protons! Protons are lost on oxidation and gained on reduction of terminal redox cofactors on the membrane opposite sides. This allows the proton transport to be reduced to the short-scale events between the outer space and these redox sites [309]. The coupling between the processes of energy supply and the reaction not only in space but also in time waits for its detailed understanding. This knowledge is highly needed to be improved for better satisfying human needs with the natural systems of photosynthesis [300] and also for creation of artificial photosynthetic machines [310], [311]. They must be capable of splitting water using the energy from the sun, an ultimate source of clean and renewable energy.

### 7.3 Cytochrome c oxidase

Cytochrome *c* oxidase (C*c*O) is the terminal enzyme complex of the respiratory chain in mitochondria and in some aerobic bacteria. In the respiratory chain, it catalyzes oxygen reduction by coupling electron and proton transfer through the enzyme with proton pumping across the membrane. Converting chemical energy into transmembrane proton gradient, it links the electron transfer from cytochrome *c* to dioxygen with proton pumping. Essentially, protons and electrons come from opposite sides of the membrane. In every catalytic cycle, CcO is able to drive eight charges from the high pH to low pH side of the membrane, increasing the electrochemical gradient. The overall reaction catalyzed by CcO is easy to write:

O_2_ + 4e^−^ + 8H^+^(*in*) → 2H_2_0 + 4H^+^(*out*).

**Fig. 27:**
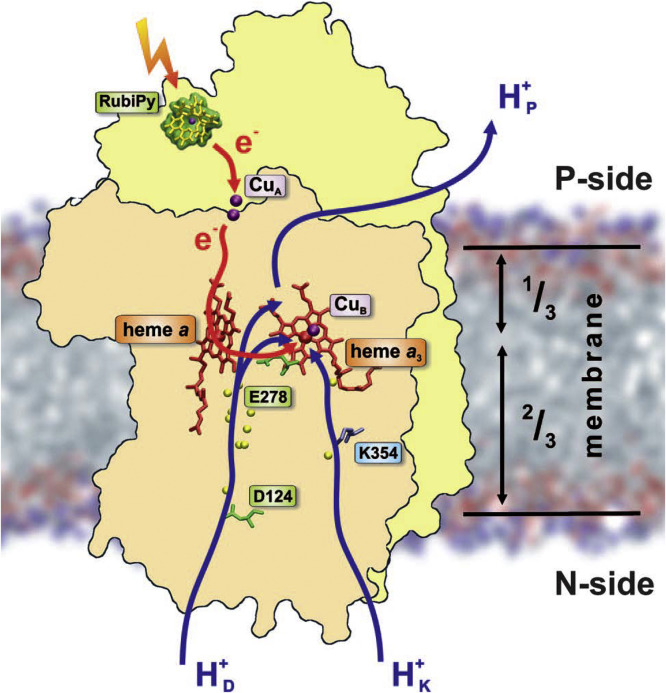
Schematic presentation of cytochrome c oxidase structure and function [313]. The two key subunits, I (pink) and II (yellow), are depicted in the membrane together with the four redox-active centers, Cu_A_, heme *a*, heme *a*_3_, and Cu_B_. Proton transfer (blue arrows) from the N-side of the membrane is of two types, H_D_^+^ and H _K_^+^, takes place via two pathways, D and K. The pumped proton H_P_^+^ is released to the P-side of membrane from the pump site above heme *a*_3_. The electron transfer path is indicated in red. The reaction can be artificially initiated by photoinduced electron injection from ruthenium bipyridyl (Ru(biPy)_3_^2+^.

The CcO reaction mechanism, however, is very complicated [312]. Four electrons and protons are used for the chemistry of exergonic oxygen reduction, while four more protons are pumped per one catalytic turnover. Thus, in this intricate machinery the protons are used in two coupled reactions, to produce water and to generate the proton gradient by its pumping across the membrane. They are taken up from the inside of the inner mitochondrial membrane (negatively charged N-side) and transferred through two specific pathways. One connects the protein surface with the catalytic site (pathway K), and the other provides the input of transported proton on N-side (pathway D). The proton, following the pathway D, is released on the opposite membrane surface (positively charged P side) (Fig. 27).

Proton uptake and proton release in CcO systems involve a number of steps of ET-PT coupling. They explore the variation of electron density as the means for dramatic influence on proton affinity. The key specific event is the proton pumping within the membrane. The driving force of this pumping must appear if the proton free energy increases to an extent that could allow its motion against the formed electrochemical gradient, which is provided by exergonic O_2_ oxidation. Proton pumping requires that some donor/acceptor couple on a pathway should dramatically change its proton affinity being able to load the low-energy protons and then supply protons with the energy high enough for operation throughout the reaction cycle. The electron supply is provided via heme *a*.

The proton motion along the pathway D is correlated with the other proton income along pathway K and with the catalytic act (water production). The fact that the two proton pathways, catalytic and pumping, do not cross and both of them are unidirectional, is essential. In addition, protons do not move as H^+^/*e*^−^ neutral hydrogen atoms: the electrons that are needed for the reaction come by quite different route starting from P side of the membrane. This means that proton motion does not proceed along the route that could minimize the electrostatic cost, which is quite different from common ET-PT reactions studied in organic photochemistry (see Section 2). Since water becoming the reaction product diffuses slowly and must be the rate-limiting step of overall process, the rates of ET and PT steps should be strongly decreased, kinetically adjusted to it.

The retardation of these steps really happens. In key experiment [313] for observing the PT reaction in real time, the laser-activated electron injection into the oxidized enzyme was used (see Fig. 27). The ET and PT reaction rates traced by applying the combination of spectroscopic and electrometric techniques were found to be unusually slow. The 10-μs ET to heme a was recorded from the attached electron donor. It raises the pK_a_ of a “pump site,” which is loaded by a proton from the inside of the membrane (pathway D) in 150 μs. This loading increases the redox potentials of both hemes, a and a_3_, which allows electron equilibration between them to proceed at the same rate. Then, in 0.8 ms, another proton is transferred from the inside to the heme a_3_/Cu_B_ center, and the electron is transferred to Cu_B_. Finally, in 2.6 ms, the preloaded proton is released from the pump site to the opposite side of the membrane.

This result is in striking contrast with simulations of PT dynamics in d-channel showing that the electron and proton motions should be faster by many orders of magnitude. The simulations provide an estimate that with the assigned free energy span of 1 eV, proton transfer should proceed in less than 100 ps [202], [226]. Such discrepancy in real and estimated rates indicates that the electron and proton transfers do not occur concertedly, they are not the ET-PT events. Even more surprising is the fact that in the coupling of exergonic (energy-releasing) O_2_ reduction to endergonic (energy-consuming) proton transfer, a strict stoichiometry is kept: for each proton consumed in the O_2_ oxidation chemistry, one proton is translocated. The coupling of these events must involve some poorly understood mechanism of gating [314]. Variation of H-bonding connectivity along the PT chain can provide the effect of such gating, but it is very hard to study this process in experiment.

**Fig. 28:**
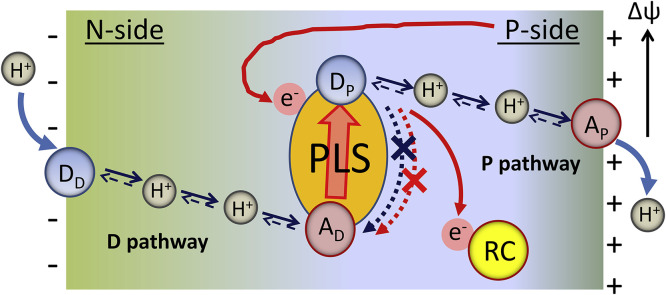
Simplified diagram illustrating possible mechanism of proton pumping in cytochrome c oxidase. d-pathway (D_D_→A_D_) and P-pathway (D_P_→A_P_) operate as proton channels translocating protons along the free energy gradients. The two pathways are connected at proton-loading site (PLS) that operates as a bi-state ‘proton lift’. In state A_D_ it operates as a proton acceptor with high proton affinity. Accepting proton, it also accepts an electron and becomes the strongly acidic site D_P_ able to donate proton. Some conformational change prevents back transition D_P_→A_D_ before proton and electron release. Electron is transferred to the reaction site (RC) and proton continues its way along the P pathway. It is released at biomembrane P-side with the free energy (Δψ) higher than that at the N-side, creating the proton gradient.

In the following discussion, we concentrate on the mechanism of proton pumping. In view that the mutations that block the proton pumping do not hamper the catalytic O_2_ reduction [315] and that blocking the catalytic proton pathway does not influence the proton pumping [316], we can provide modeling the pump-related moiety as a separate sub-system. It is essential here to account the presence of one proton – one electron stoichiometry, which immediately suggests the proton transfer gating by electron motion [317]. The way, how this is done can be suggested from the known facts that by accepting an electron and becoming an electron donor, the system also can become a strong proton donor. An analogy can be drawn with ‘super photoacids’ in photochemistry: the light absorption by protonated chromophore, redistributing the electron density, may decrease the pK_a_ values by many orders of magnitude [286], [287]. (Strictly, the pK_a_ measures cannot be applied if proton dissociation/association proceeds not in water). This dramatic modulation of proton affinity by electron transfer may be the essence of operating the proton-loading site (PLS) that is thought to be central in the mechanism of proton pumping in CcO [318], [319], [320]. This effect is probably similar to that observed for bacteriorhodopsin photocycle (Section 7.1). A simple model illustrates how this can be done (Fig. 28).

It can be assumed that the two sections of proton route to and from PLS, the d-pathway (D_D_→A_D_) and P-pathway (D_P_→A_P_), are the proton wires that operate in a similar way to other proton channels discussed in this paper. They are formed by H-bonds connecting proton donors and acceptors and translocate protons along the free energy gradients formed due to gradients in proton affinities of donors and acceptors. In this way, like in other channels considered above, the unidirectional proton flow proceeds with proton passing one-by-one coupled with PLS cycle, and the back transfer is suppressed.

The key element here is the ‘proton lift’ of PLS that can switch with the electron uptake and proton release between two regimes – protonated but electron-free state (as acceptor A_D_) and deprotonated but electron-bound state (as donor D_P_). When A_D_ binds proton, the whole D_D_→A_D_ pathway for entering of new protons becomes blocked. Its electron-induced transition to D_P_ pumps proton to D_P_→A_P_ channel. Then in PLS the reverse transition should occur for the new cycle to begin, but on a condition of blocking the reverse transfer of proton and electron. Without electron, the site becomes again A_D_ ready to accept another proton. The electron can be transferred to the O_2_ reducing reaction site that should have higher electron affinity. Thus, strictly one electron is needed for pumping one proton. A conformational change, possibly one of those described earlier [321], triggered by electron leaving its location site at heme *a*, can play this role.

This simple scheme looks reasonable and compatible with experimental and computational results. Some structural data with assignment of heme and copper sites that can be used for its background can be found in review [322] and more recent numerous experimental and computational papers [323]. Based on this scheme, gating of protons by electrons based on strict stoichiometry of electrons that are delivered and protons transferred [322] becomes easily conceivable. Proposed by other authors [314], [324], [325], [326] more complicated schemes, such as involving a second proton, several potential barriers or extended conformational changes in protein, may not be needed. The frequently discussed strict directionality of proton flow [327] comes out naturally from the present model assuming that both D_D_→A_D_ and D_P_→A_P_ are the common (see above) proton channels [328] guided by donor-acceptor electrostatic field gradients. These channels were identified recently in CcO protein structure as being the water wires [329].

Concluding the Section on bioenergetics, one of the recent papers [314] can be cited. “Concerning the nature of the gates forcing the protons to move against the electrochemical gradient, it should be noted that most of the suggested pumping mechanisms actually do not even have suggestions for these gates.” The reader may note that in a presented above simple picture describing PT dynamics in CcO, PSII and BR the ‘proton gate’ is substituted by a ‘proton lift’ located at PLS site and possessing the function of raising the proton energy. The similarities between reaction mechanisms found for formation of proton gradient in these systems are not circumstantial but are the reflections of a general principle.

Guided by this principle, we come to a general conclusion on a key distinctive feature of proton pumps from proton channels. Unidirectional flow in channels and channel segments of pumps can be realized by quite similar means. Special is the participation of ‘proton lift’ possessing the following characteristics: (1). It is a single localized group of atoms pumping the energy into transiently charged/recharged proton donor/acceptor site. (2). Recharging is guided by transient transfer of electronic charge followed by repolarization of the local protein environment and conformational changes. The ET and PT pathways can be separated and follow their own mechanisms and routes. (3). The ‘proton lift’ does not need to contact with primary donor or terminal acceptor directly. The connections are via the channel segments.

## 9 Conclusions and prospects

The future of biochemistry is seen as a strong move from sole description of the basic molecular transformations essential for life towards their explaining and then to predicting and exploring them in modern technologies. To date, innumerable amount of protein structures is resolved to atomic level and their role in biological systems assigned. Directed mutagenesis, the use of inhibitors, chemical modifications and other tools allowed establishing the involvement of different groups forming the active sites, proton channels and beyond [330], [331]. Still, synthetic protein analogs remain to be less efficient as catalysts by many orders of magnitude, and the technologies exploring the biomembrane principle of selectivity are lacking. A lot of effort is observed to mimic the biological charge transfer systems in design of photovoltaic devices [332], artificial photosynthesis [333] and bioelectronics [334], meantime the progress is slow. All that means that some important issues are missing or underestimated. In author's opinion, this is because of the lack of understanding of the basic events of transfer of elementary charges, electrons and protons in biological structures. Thus, we observe the great gap between the descriptions of what is happening in biological ET-PT reactions provided by structural data and the explanations how this happens based on the physical analysis of observed phenomena. The X-ray and neutron crystallography [335], [336], site-directed mutagenesis [330], electrochemical methods, as well as DFT calculations [47] provide important but insufficient information on these basic events.

On the other hand, the studies of ET-PT reactions presently suggested by organic photochemistry can only partially satisfy the researchers in analyzing the biochemical reaction mechanisms. Indeed, they provide the basic principles, but regarding real systems, they are presently considered of value only on a conceptual level. The ability to provide in photochemical experiment the input of energy in a short light pulse synchronizing the population of reactants should be used in full to observe the reaction development with high resolution in time and energy. This possibility should be realized in new systems approaching closer the real functional behavior.

Addressing the necessary level of complexity needs new ideas and their employment in new ingeniously designed model systems. They can be simplified and contain only the active chemical components necessary for ET-PT and function-related influences for obtaining detailed information about the fundamental properties that govern these reactions. Organic ET-PT systems described in the first part of this Review can be used as the prototypes of their building blocks. Particular requirements for these future developments may be pointed to the following issues:

- The coupling of multiple reaction steps that could allow unidirectional flow of reactants in the direction of products, avoiding high-energy intermediates and realizing the correlation between individual steps on time and energy scales with particular emphasis on the PT driving force operating at long distances. Realization of kinetic traps and of local (in the absence of global) thermodynamic equilibria [337].
- The finding of model systems that allow the proton and electron motions to be orthogonal, so that the proton and electron transfer distances and driving forces may be independently controlled and used as the parameters in studied models [7].
- The demonstration of different aspects of modulating role of conformational changes and charge rearrangements on PT donor or acceptor reactivity, particularly in the case of their distant location [11].
- The modeling of effects of pre-organization in reaction media by locating and orienting of charges and dipoles in a specific pattern [338], simulating specific enzyme structures with their correctly oriented electric fields created by constituting atomic groups [161].
- The possibility of variations in molecular rigidity and functionally oriented molecular dynamics possessing hierarchical character and proceeding on different time and length scales [339]. Comparison of performance of PT relays in liquid and solid systems in order to evaluate the role of diffusional motions and thermal fluctuations that activate or destroy the ET-PT network. Motions of water molecules in the performed channels are of particular interest [340].

This currently very active area at an interface of chemistry and biology offers many possibilities for detailed kinetic and mechanistic description of ET-PT processes in different enzymes, proton transporters and pumps. It does not only suggest an exciting challenge in explaining hitherto obscure phenomena, but also stands to provide a picture of the basic chemical requirements necessary to design better catalytic and charge transporting systems.

## Funding

This research has received no external funding.

## Declaration of Competing Interest

I am the author of submitted paper “Proton transfer reactions: from photochemistry to biochemistry and bioenergetics” and I declare that this paper was written solely by me on my own initiative and no financial support was received on its publication.

## Data Availability

No data was used for the research described in the article.
